# Nitrogen-Fixing Bacteria Promote the Growth of *Fritillaria taipaiensis* P. Y. Li by Regulating Physiological and Biochemical Reactions and Protecting Enzyme System-Related Gene Expression

**DOI:** 10.3390/biology14040325

**Published:** 2025-03-24

**Authors:** Mingyan Ye, Jiaqi Lang, Xiaotian Kong, Zhifen Shi, Fengjie Duan, Guiyong Qin, Hua Zhang, Dongqin Guo, Nong Zhou

**Affiliations:** 1 College of Biological and Food Engineering, Chongqing Three Gorges University, Chongqing 404120, China; cqyydye@163.com (M.Y.); lang1jiaqi@163.com (J.L.); duanfengjie07@163.com (F.D.); zhanghua03129@163.com (H.Z.); 2 College of Agriculture and Forestry Science and Technology, Chongqing Three Gorges Vocational College, Chongqing 404155, China; qinguiyonghaode@163.com; 3 College of Pharmacy, Dali University, Dali 671000, China; kongxiaotian156@163.com; 4 College of Modern Chinese Medicine Industry, Chengdu University of Traditional Chinese Medicine, Chengdu 611130, China; shizhifen@stu.cdutcm.edu.cn; 5 School of Pharmacy, Chongqing Three Gorges Medical College, Chongqing 404120, China

**Keywords:** antioxidant enzymes, gene expression, nitrogen-fixing bacteria, plant physiology and chemical reactions, Chinese medicinal material

## Abstract

The widespread application of chemical fertilizers and pesticides has resulted in environmental pollution. With the growing emphasis on ecological agriculture in traditional Chinese medicine, microbial fertilizers are increasingly recognized for their potential. The aim of this study is to investigate the effect of inoculating nitrogen-fixing bacteria on the soil (yellow loam, river sand, and organic fertilizer in a 2:1:1 ratio) of *Fritillaria taipaiensis*, with a focus on the leaf changes in terms of physiological parameters, antioxidant enzyme activity, and corresponding gene expression levels. The experiment involved three nitrogen-fixing bacteria, namely *Rahnella aquatilis*, *Pseudomonas chlororaphis*, and *Paenibacillus stellifer*, with a total of eight treatment groups. The objective was to assess how these bacterial treatments influenced physiological parameters, photosynthetic characteristics, pigment content, and both antioxidant enzyme activities and gene expression in the leaves of *F. taipaiensis*. The experimental results demonstrated statistically significant reductions (*p* < 0.05) in malondialdehyde (MDA) content and stomatal limitation value (LS) in *F. taipaiensis* leaves under treatment conditions relative to the control group (CK). The most substantial decreases were observed dual-inoculation with *R. aquatilis* and *P. stellifer* (N5), showing reductions of 38.24% and 20.94% in MDA and LS compared to CK values. Additionally, leaf area, leaf thickness, stem thickness, plant height, photosynthetic parameters, pigment content, soluble sugars, soluble proteins, proline levels, and the activities of superoxide dismutase (SOD), peroxidase (POD), and catalase (CAT) exhibited varying degrees of increase. Compared to the CK group, the SOD, POD, and CAT activities of the N5 group increased by 141.06%, 160.59%, and 106.23%, respectively. The relative gene expression patterns of SOD, POD, and CAT corresponded with the trends observed in their respective antioxidant enzyme activities. Pearson correlation analysis further demonstrated that leaf area and net photosynthetic rate (Pn) were significantly correlated with respect to SOD, POD, and CAT activities, as well as their corresponding gene expression levels. In conclusion, inoculation with nitrogen-fixing bacteria improved the growth and stress tolerance of *F. taipaiensis*, with the combined application of *Rahnella aquatilis* and *Pseudomonas stellifer* yielding the most effective results. This study establishes that different rhizosphere nitrogen-fixing bacteria, either individually or in combination, influence the photosynthetic characteristics, physiological and biochemical parameters, and protective enzyme systems of *F. taipaiensis*. These findings provide a theoretical foundation for the selection of nitrogen-fixing bacteria as biofertilizers in the artificial cultivation of *F. taipaiensis* and highlight their potential application in the cultivation of traditional Chinese medicinal materials.

## Simple Summary

To reduce the influence of chemical fertilizers and pesticides on the cultivation of *Fritillaria taipaiensis* P. Y. Li, this study adopted the application of microbial fertilizer to mitigate soil damage and enhance the plant’s stress resistance. In this experiment, the growth index, enzyme activity, and gene expression of *F. taipaiensis* leaves were measured by applying nitrogen-fixing bacteria. The results showed that nitrogen-fixing bacteria could promote the growth and development of *F. taipaiensis*. This study not only provides a theoretical foundation for the subsequent cultivation technology of *F. taipaiensis* but also provides a new idea in terms of the realization of green planting of Chinese medicinal materials.

## 1. Introduction

*Fritillaria taipaiensis* P. Y. Li is a perennial herbaceous plant of medicinal value belonging to the genus *Fritillaria* within the family Liliaceae. Traditionally, it has been utilized in the treatment of lung meridian ailments [1]. The wild populations of this species have encountered significant damage, approaching a state of depletion, primarily owing to extensive human excavation and subsequent destruction of the surrounding ecological habitat [2]. In response to the impending scarcity of this resource, the Chinese Pharmacopoeia has incorporated the dried bulb of *F. taipaiensis* as an acceptable cultivated alternative to *F. cirrhosa* D. Don [3]. The historical record of cultivating *F. taipaiensis* can be traced to the “Daning County Annals” [4], wherein it is proclaimed that “*F. taipaiensis*, produced in Yinchangping, is the best”; the term “Daning County” presently refers to Wuxi County in Chongqing City. Subsequently, domestication of wild *F. taipaiensis* was undertaken in Shaanxi, Sichuan, and Chongqing provinces [5,6,7].

With the excessive use of fertilizers, the Ministry of Agriculture and Rural Affairs has issued the “Action Plan for Chemical Fertilizer Reduction by 2025” [8], emphasizing the urgency to expedite the substitution of chemical fertilizers with organic fertilizers and promote the use of microbial fertilizers [9]. Continuous cropping obstacles and soil compaction, resulting from the extensive use of chemical fertilizers and pesticides in agricultural production, pose a significant threat to the quality of medicinal plants and soil health. However, the emergence of ecological agriculture principles within traditional Chinese medicine (TCM) cultivation has positioned microbial fertilizers as a strategic response to chemical fertilizer reduction mandates. These biofertilizers concurrently enhance medicinal plant yield and quality, preserve soil ecological integrity, and propel sustainable industrialization of TCM agroecosystems [10].

The screening of highly efficient plant growth-promoting bacteria constitutes a fundamental prerequisite for microbial fertilizer development [11]. Xiucheng Wang [12] identified *Herbaspirillum seropedicae* DX35, an endophytic nitrogen-fixing bacterium residing in rice root systems, which demonstrates multifunctional agricultural potential through its phosphate-solubilizing capacity and siderophore secretion capabilities. This strain biosynthesizes indole-3-acetic acid (IAA), a phytohormone that enhances root system development and facilitates nutrient absorption efficiency in rice plants. Associative nitrogen-fixing bacteria establish mutualistic symbiosis with sugarcane crops. These diazotrophic microorganisms exhibit a tripartite growth-promoting mechanism encompassing biological nitrogen fixation, inorganic phosphate solubilization, and phytohormone production, collectively contributing to enhanced plant growth and development [13].Additional investigations have revealed that the external inoculation of growth-promoting bacteria, in symbiosis with the root system of *F. taipaiensis*, can foster an advantageous arbuscular mycorrhizal structure, thus enhancing nutrient accumulation in medicinal materials [14].

Plant growth is fundamentally linked to the administration of nitrogen-containing fertilizers, the predominant source of which is synthetic industrial ammonia. This synthetic process is highly energy-intensive and contributes to greenhouse gas emissions [15], raising environmental concerns. Conversely, biological nitrogen fixation is a cost-effective alternative that is less prone to pollution through volatilization, denitrification, or leaching loss [16]. Biological nitrogen fixation enables the fixation of 100 million tons of nitrogen fertilizer annually [17], signifying its promising potential in cultivation practices. Biological nitrogen fixation relies on nitrogen-fixing bacteria, which convert atmospheric nitrogen into a nitrogen fertilizer that plants can directly utilize and supply for their growth and development [18]. Furthermore, plant growth is intricately connected to photosynthesis, a process whereby light energy is harnessed via chloroplasts to transform carbon dioxide and water into energy-storing organic matter while releasing oxygen. Within this process, chlorophyll functions as the principal agent of light energy absorption, conversion, and transmission; its content notably influences the photosynthetic rate [19]. Research has demonstrated that the application of microbial fertilizers can enhance chlorophyll accumulation [20], augment photosynthetic enzyme activity [21], and expedite the rate of photochemical reactions within plants [22].

When plants are damaged by the external environment, compounds such as malondialdehyde (MDA) and proline (Pro) act to mitigate or minimize physiological damage, while soluble sugar and soluble protein function in osmotic regulation, providing energy to organisms and enhancing their stress resistance [23]. MDA, a byproduct of lipid peroxidation in plants, is an indicator of cell membrane damage, and inversely its levels relate to stress resistance in plants [24].

Elevated SOD activity is frequently advantageous for plants inhabiting low-nitrogen environments [25]. In a drought stress-induced environment, Jiali Xie et al. [26] utilized “Longshu 7” as a research subject and potted plants as test material. By controlling water supply and applying varying amounts of potassium fertilizer, they demonstrated that leaf enzyme activity (superoxide dismutase (SOD), peroxidase (POD), catalase (CAT)) and *Solanum tuberosum* L. tubers yield could be improved. As indexes of stress resistance, enzymes such as SOD, POD, and CAT play pivotal roles in neutralizing reactive oxygen species and free radicals [27].

At present, the body of research concerning the impact of nitrogen-fixing bacteria inoculation on the physiological indexes and antioxidant capacity of *F. taipaiensis* leaves remains limited, so this study sought to address this by applying nitrogen-fixing bacteria isolated from the rhizosphere soil of *F. taipaiensis* to its cultivation process. In this study, the assessment was carried out through the measurement of various parameters, including the leaf area, photosynthetic characteristics, content of photosynthetic pigments, activities of antioxidant enzymes, and alterations in the leaf physiological index pertaining to *F. taipaiensis* leaves. The objective of this inquiry was to discern the influence of nitrogen-fixing bacteria inoculation on *F. taipaiensis* and to ascertain the most efficacious inoculation procedure. This empirical approach is aimed at augmenting existing cultivation techniques and furnishing a foundational basis for subsequent experiments. 

## 2. Materials and Methods

### 2.1. Strain Isolation and Identification

Based on a literature review [28,29], the main distribution areas of *F. taipaiensis* were in Shaanxi, Hubei, Gansu, Chongqing, Sichuan, and other provinces. The rhizosphere soil of *F. taipaiensis* was collected from Chongqing, Sichuan, Shaanxi, Hubei, and Yunnan regions. The nitrogen-fixing bacteria were isolated from the rhizosphere soil by the dilution-coating plate method [30]. The isolated bacteria were cultured in a nitrogen-free medium (10 g mannitol, 0.2 g KH_2_PO_4_, 0.2 g MgSO_4_·7H_2_O, 0.2 g NaCl, 0.1 g CaSO_4_·2H_2_O, 5 g CaCO_3_, 20 g agar, and 1000 mL distilled water) in an incubator at 28 °C for 3 days. The purified strains were stored at −80 °C.

Gene phylogenetic analysis: The full-length 16S rDNA gene was amplified via polymerase chain reaction (PCR) using universal bacterial primers 27F (5′-AGAGTTTGATCCTGGCTGAG-3′) and 1492R (5′-TACGGCTACCTTGTTACGACTT-3′). Size-fractionation of the PCR products on a 1.3% agarose gel revealed a distinct amplified product of approximately 1500 bp. Amplified products were sequenced by Shenggong Biotechnology (Chengdu) Co., Ltd. (Chengdu, China). The obtained sequences were subjected to homology analysis using the BLAST (https://blast.ncbi.nlm.nih.gov/) algorithm against the GenBank nucleotide database. Phylogenetic reconstruction was performed with MEGA 7.0 software. A phylogenetic tree was constructed using the p-distance model, with 1000 bootstrap replicates.

The strains of nitrogen-fixing bacteria were preliminary screened using a nitrogen-fixing ring [31]. Nitrogen-fixation efficiency and indole-3-acetic acid (IAA) production capacity of the strain were determined by liquid fermentation [32] and Salkowski reaction [33]. Three dominant nitrogen-fixing bacteria were comprehensively screened out by using the entropy weight method [34]. We have previously accomplished these tasks, and detailed information can be obtained from a previous paper [33].

### 2.2. Test Site and Experimental Design

The experimental site was situated in Hongchi Dam, Wuxi County (31°38′8″ N, 108°56′30″ E), China. It features a subtropical warm and humid monsoon climate, sits at an elevation of approximately 1899 m, and has an average annual temperature of 13 °C, with an average annual precipitation that ranges from 1030 to 1950 mm. Moreover, the annual average sunshine duration is 1580 h. The test soil comprising yellow loam, river sand, and organic fertilizer (2:1:1) was obtained from Chongqing Three Gorges University. The fundamental physicochemical properties were as follows: soil pH, 7.52; organic matter content, 43.36 g/kg; total nitrogen content, 0.33 g/kg; alkaline nitrogen content, 37.88 mg/kg; available phosphorus content, 22.29 mg/kg; and available potassium content, 288.34 mg/kg.

*F. taipaiensis* bulbs were cultivated in 1.5-gallon plastic pots measuring 19.8 cm in outer diameter, 18 cm in base diameter, and 20 cm in depth. Prior to use, the pots were disinfected with 75% ethanol and allowed to dry. Eight discrete treatments were established for this experiment (see Table 1 for detailed information). In October 2022, *F. taipaiensis* bulbs were transplanted into pots for experimental purposes, with five bulbs per pot (total fresh weight approximately 1.5 g). In March 2023, bacterial strains were inoculated into a sterile beef extract–peptone liquid medium and cultured at 180 rpm for 2 days, individually. Then they were diluted with sterile water and the bacterial content was adjusted to approximately 1 × 10^8^ CFU/mL. Subsequently, bacterial fertilizer was applied via a drenching method directly to the root zone of *F. taipaiensis* plants under controlled conditions at 18 °C. After a 40-day period, leaf samples from *F. taipaiensis* were harvested for analysis.

### 2.3. Index Measurement

Assessment of growth indices with respect to *F. taipaiensis* leaves: In this experiment, ten potted plants under sunny weather conditions were selected from each parallel group, and the parameters of one plant in each pot was measured. Parameters such as leaf area, height, stem diameter, and leaf thickness were measured using either a standard ruler or a vernier caliper. The leaf area was ascertained utilizing the length–width ratio method [35].

Analysis of photosynthetic and pigment content in *F. taipaiensis* leaves: At an external CO_2_ concentration of 400 μmol/mol, the net photosynthetic rate (Pn), stomatal conductance (Cond), intercellular CO_2_ concentration (Ci), and transpiration rate (Tr) of *F. taipaiensis* leaves were evaluated using a photosynthesis analyzer (LI-6400, LI-COR, Inc., Lincoln, NE, USA) from 10:00 a.m. to 12:00 p.m. on 1 May. The stomatal limitation values (LS) were calculated according to the method described by Farquhar and Sharkey [36], and the content of photosynthetic pigments was determined using the methodology of Zhiliang Zhang [37].

Quantification of MDA, soluble sugar, soluble protein, and proline in *F. taipaiensis* leaves: In this experiment, six potted plants under sunny weather conditions were selected from each parallel group, and the indexes of one plant in each pot were measured. The concentrations of malondialdehyde (MDA) and soluble sugar were quantified by employing the thiobarbituric acid technique [38]; the soluble protein content was ascertained by the coomassie blue staining methodology [39]; and the proline content was measured by the ninhydrin method [40].

Evaluation of antioxidant enzyme activity in *F. taipaiensis* leaves by referencing the methodology of Kun Ge et al.: Six potted plants under sunny weather conditions were selected from each parallel group, and the indexes of one plant in each pot were measured. SOD activity was gauged through the nitrogen blue tetrazolium method [41]; POD activity was determined by the guaiacol chromogenic method [41]; and CAT activity was evaluated with ultraviolet spectrophotometry [41].

Analysis of gene expression associated with the antioxidant enzyme system in *F. taipaiensis* leaves: Total RNA was extracted from *F. taipaiensis* leaves using the TRIzol^®^ Plus RNA Purification Kit. (Thermo Fisher Scientific, Waltham, MA, USA). Its content, purity, and quality were assessed by ultraviolet spectrophotometry and gel electrophoresis, with an optical density value (A260/A280) requirement of 1.8–2.0. RNA was reverse transcribed into cDNA using the SuperScript™ III First-Strand Synthesis Super Mix for qRT-PCR Kit (Thermo Fisher Scientific, Waltham, MA, USA), following a 25 °C incubation for 10 min, a 50 °C incubation for 30 min, and an 85 °C incubation for 5 min. The cDNA was stored at −20 °C for later use. Primer Premier 6.0 and Beacon Designer 7.8 software were utilized to create RT-qPCR primers (Table 2), synthesized by Nanjing Aoqing Biotechnology Co., Ltd. (Nanjing, China), employing the *rp116* gene (Accession Numbers: MG525382.1) as an internal reference [42]. The fluorescence quantitative reaction system comprised 10 µL of 2 × Taq Pro Universal SYBR qPCR Master Mix, 1 μL of cDNA template, 0.4 μL each of forward and reverse primers, and an appropriate amount of water, with a total volume of 20 μL. The reaction conditions were as follows: 95 °C for 2 min, 95 °C for 20 s, and 58 °C for 20 s, for 39 cycles. A melting curve was also plotted, each sample was analyzed in triplicate, and the relative expression levels were calculated using the 2^−∆∆Ct^ method [43].

### 2.4. Statistical Analysis of Data

Data processing and organization were conducted using Microsoft Excel 2010 (Microsoft Corp., Redmond, WA, USA), followed by statistical analyses performed in SPSS 24.0 (IBM Corp., Armonk, NY, USA). Graphical visualizations were generated with Origin Pro 2021 (OriginLab Corp., Northampton, MA, USA). Pearson correlation coefficients were calculated to assess linear relationships between variables. Single-factor analysis of variance (ANOVA) was applied with a 95% confidence interval (α = 0.05) to determine statistical significance.

## 3. Results

### 3.1. Strain Isolation and Screen

**Figure 1:**
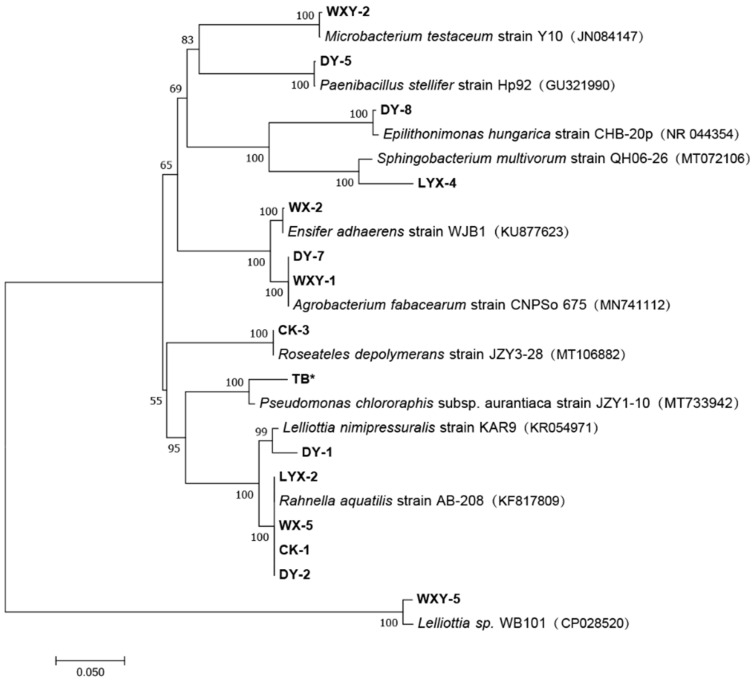
Phylogenetic tree based on the 16S rDNA gene sequence. Note: numbers denote NCBI Accession Numbers; the scale bar denotes phylogenetic distance. * denotes a type of marker symbol, no functional implications.

Fifteen strains of nitrogen-fixing bacteria were identified in the rhizosphere soil of *F. taipaiensis* (Figure 1). Their 16S ribosomal RNA gene sequences are detailed in Appendix A, Table A1. Phylogenetic analysis revealed that the 15 strains were taxonomically classified into 10 distinct genera spanning nine bacterial families. Notably, strains DY-7 and WXY-1 clustered within the *Agrobacterium fabacearum* clade, while strains LYX-2, WX-5, CK-1, and DY-2 showed close phylogenetic affinity to *Rahnella aquatilis*.

The relevant growth-promoting indicators are presented in Table 3, while the detailed process for calculating nitrogen-fixation efficiency values is comprehensively described in Appendix A, Table A2. The nitrogen-fixing efficiency of the 15 strains ranged from 0.38 to 8.49 g/kg. Compared to the non-inoculated seed culture, WX-5 (*Rahnella aquatilis)* had the best ability at 8.49 g/kg, *Pseudomonas chlororaphis* was capable of 2.23 g/kg, and *Paenibacillus stellifer* was capable of 2.57 g/kg. Compared to the non-inoculated medium, *Rahnella aquatilis*, *Pseudomonas chlororaphis*, and *Paenibacillus stellifer* increased indole-3-acetic acid (IAA) levels by 7.42, 10.12, and 2.29 µg/mL, respectively.

The comprehensive scores for *Rahnella aquatilis*, *Pseudomonas chlororaphis*, and *Paenibacillus stellifer* were 0.604, 0.494, and 0.470, respectively. The three strains secured first, third, and fifth positions in the composite ranking based on multiple growth-promoting indicators. Therefore, these three nitrogen-fixing bacteria were used in the preparation of bacterial fertilizer applied to *F. taipaiensis*.

### 3.2. Influences of Different Treatments on the Growth Index of F. taipaiensis Leaves

As delineated in Table 4, compared to the control (CK) group, distinct treatments were observed to augment the leaf area, leaf thickness, stem thickness, and plant height of *F. taipaiensis*, which was statistically significant (*p* < 0.05). Compared to the CK group, in the single-inoculation treatment groups (N1, N2, N3), no statistically significant differences of their effects on leaf area, stem diameter and plant height were observed. In the dual-inoculation treatments (N4, N5, N6), the N5 group exhibited a 61.11% increase in leaf area and a 73.45% increase in plant height compared to CK. Similarly, the N6 group showed a 96.25% enhancement in leaf thickness and a 28.44% increase in stem diameter. In the triple-inoculation treatment group (N7), the stem diameter was 1.29 times that of the CK group, an increase of 29.47%. Notably, dual-inoculation treatments outperformed both single-strain and triple-strain inoculation treatments, with the dual-inoculation of *R. Aquatilis* and *P. stellifer* (N5) demonstrating optimal growth promotion in *F. taipaiensis*.

### 3.3. Influences of Different Treatments on Photosynthetic Parameters and Photosynthetic Pigment Content of F. taipaiensis Leaves

Table 5 shows the influences of diverse treatments on the photosynthetic parameters of *F. taipaiensis* leaves. Distinct treatments were observed to significantly increase net photosynthetic rate (Pn), stomatal conductance (Cond), intercellular CO_2_ concentration (Ci), and transpiration rate (Tr) values (*p* < 0.05). In contrast with the CK group, in the single-inoculation treatment groups, the Pn increase in the N2 treatment group was the most significant, at 20.21%, while Cond, Ci, and Tr increases in the N3 group were 1.16-fold, 1.22-fold and 1.16-fold those of the CK group, increasing by 16.18%, 21.72%, and 15.85%, respectively. Among the dual-inoculation treatment groups, the N5 group proved to be the most effective, augmenting the Pn, Cond, Ci, and Tr by 29.83%, 61.76%, 46.87%, and 41.93%, respectively, compared to the CK group. Notably, dual-inoculation treatments outperformed both single-strain and triple-strain inoculation treatments, with the dual-inoculation of *R. Aquatilis* and *P. stellifer* (N5) demonstrating optimal growth promotion in *F. taipaiensis*.

Table 6 shows the levels of photosynthetic pigments in the *F. taipaiensis* leaves. In juxtaposition with the control group, chlorophyll a, chlorophyll b, and total chlorophyll content were significantly increased across treatments (*p* < 0.05). The chlorophyll a content of the treatment groups ranged from 0.82 to 1.02 mg/g, with a 7.91% increase observed in the single-inoculation treatment group (N1) and the largest increase observed in the dual-inoculation treatment group (N5), with a 10.62% increase relative to CK. The chlorophyll b content ranged from 0.24 to 0.35 mg/g, with the highest increase of 44.26% observed in the single-inoculation treatment group (N2) and 49.36% observed in the N5 group relative to CK. Expect for the N5 group, the carotenoid content of the other treatments was observed to be lower than that of the CK group. The total chlorophyll content ranged from 1.15 to 1.37 mg/g, with a 15.63% increase in the N1 group and the largest increase of 18.48% in the N5 group relative to CK. Notably, with the exception of N5, the carotenoid content of the treatments was lower than that of CK. Diverse treatments were capable of elevating the content of photosynthetic pigments in *F. taipaiensis* leaves, with the N5 group yielding the most pronounced effect.

### 3.4. Influences of Different Treatments on MDA, Soluble Sugar, Soluble Protein, and Proline Levels of F. taipaiensis Leaves

As shown in Table 7, the malondialdehyde (MDA) content of *F.taipaiensis* leaves was significantly lower in the treatment groups than in the control (CK) group, while the concentrations of soluble protein and proline (Pro) were found to be significantly higher compared to the CK group (*p* < 0.05). All treatment groups exhibited lower MDA content compared to the CK group, with the dual-inoculation treatment group (N5) showing the most significant reduction (38.24%). In single-inoculation treatment groups, the N3 treatment enhanced soluble sugar and soluble protein content by 36.98% and 61.24%, respectively. Notably, the N5 group exhibited a 113.45% increase in soluble protein content relative to the CK group. Proline content in the triple-inoculation group (N7) reached a 1.72-fold increase with respect to CK levels, representing the highest elevation (71.64% increase) among all treatments.

### 3.5. Influences of Different Treatments on Antioxidant Enzyme Activities in the Leaves of F. taipaiensis

**Figure 2:**
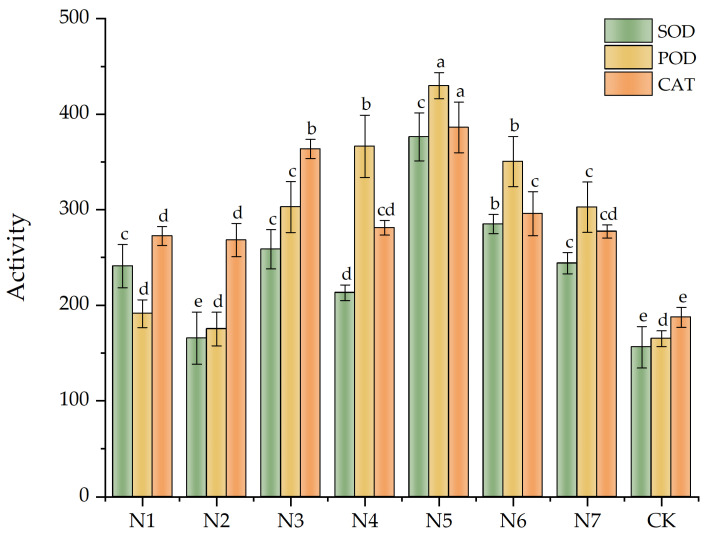
Effects of antioxidant enzyme activities on *Fritillaria taipaiensis* P. Y. Li leaves under various treatments. Note: Within the same color group, differing lowercase letters denote statistically significant differences at the *p* < 0.05 level as determined by Fisher’s Least Significant Difference (LSD) post hoc test. Letter assignments follow a descending order of significance, where ‘a’ indicates the most pronounced difference, followed sequentially by ‘b’, with subsequent letters (c, d, etc.) representing progressively smaller magnitudes of divergence.

As shown in Figure 2, specific numerical values were documented in Appendix A, Table A3 and Table A4. The protective enzyme activity values were higher in all treatment groups compared with the CK group. Upon analyzing all the data, we noted that the SOD activity enhancement effect in the dual-inoculation treatment group (N5) was the best, followed by the N6 group. This effect was approximately 141.06% and 82.55% higher in the N5 and N6 groups than in the CK group, respectively. The POD enhancement effect in the N5 group was the best, which was approximately 160.59% higher than that of the CK group, followed by the N4 and N3 groups. The CAT activity enhancement effect in the N5 group was the most obvious, which was 106.23% higher than that of the CK group. The dual-inoculation treatment groups more significantly improved the protective enzyme activity. The SOD, POD, and CAT activities of dual-inoculation treatment group N7 were comparable to those observed in single-inoculation groups, with no significant difference detected (*p* > 0.05).

### 3.6. Influences of Different Treatments on Genes Related to Antioxidant Enzyme Systems in the Leaves of F. taipaiensis

**Figure 3:**
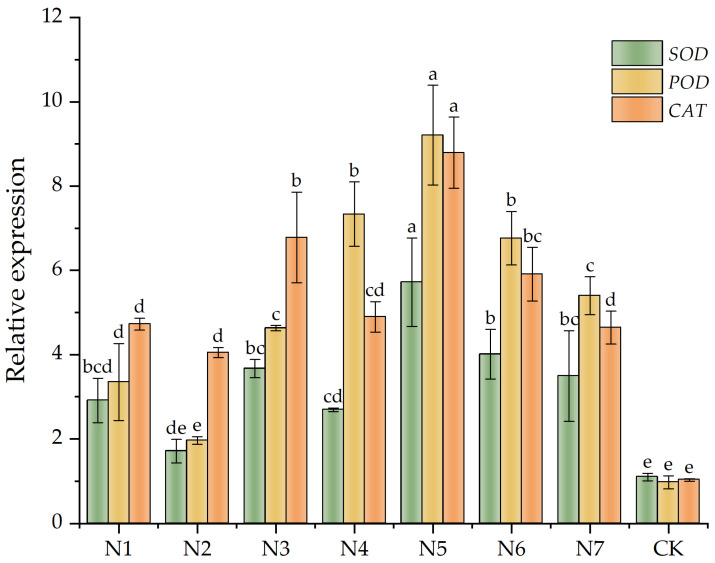
SOD, POD, and CAT genes related to the protective enzyme systems in leaves of *Fritillaria taipaiensis* P. Y. Li. Note: Within the same color group, differing lowercase letters denote statistically significant differences at the *p* < 0.05 level as determined by Fisher’s Least Significant Difference (LSD) post hoc test. Letter assignments follow a descending order of significance, where ‘a’ indicates the most pronounced difference, followed sequentially by ‘b’, with subsequent letters (c, d, etc.) representing progressively smaller magnitudes of divergence.

RT-qPCR analysis revealed significant variations in the relative mRNA levels of *SOD*, *POD*, and *CAT* genes within the leaves of *F. taipaiensis* across distinct treatments (Figure 3), with specific numerical values documented in Appendix A, Table A5. The gene expression levels of *SOD*, *POD*, and *CAT* increased in all treatment groups compared with the CK group. The dual-inoculation treatment group N5 exhibited the most pronounced elevations, with *SOD*, *POD*, and *CAT* mRNA levels reaching 5.20-fold, 9.47-fold, and 8.52-fold increases with respect to CK values, respectively. In contrast, the dual-inoculation group N7 showed expression levels comparable to single-inoculation groups, with no statistically significant differences detected (*p* > 0.05). These findings indicate that dual-inoculation treatments exert differential modulatory effects on antioxidant gene expression, with the N5 group demonstrating the highest transcriptional activation levels among all experimental groups.

### 3.7. The Pearson Correlation Analysis

**Figure 4:**
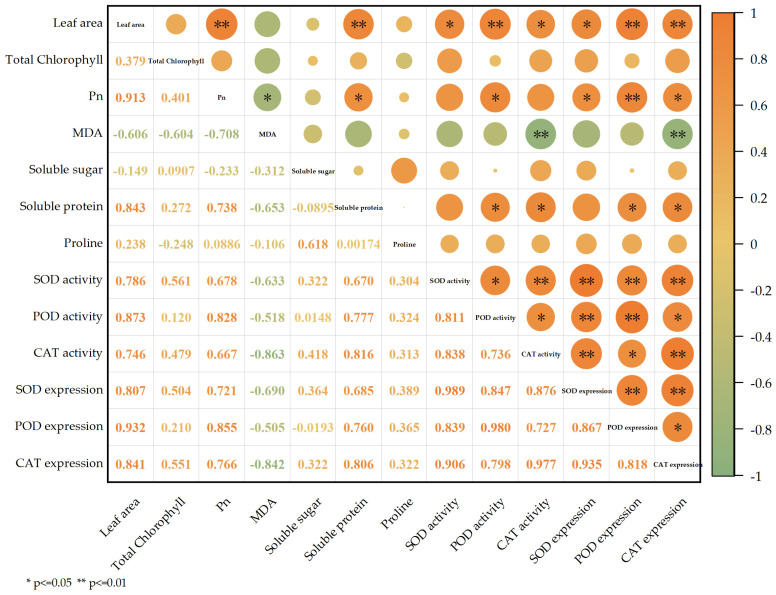
The Pearson correlation analysis between different indicators. Note: Orange in the figure indicates a positive correlation, green indicates a negative correlation, and numbers indicate correlation coefficients. * denotes *p* ≤ 0.05, ** denotes *p* ≤ 0.01.

In this study, the Pearson correlation analysis was performed on the leaf area; total chlorophyll, Pn, MDA, soluble sugar, soluble protein, and free proline content; the activity index of the three protective enzymes; and the relative gene expression levels of the three protective enzymes (Figure 4). Notably, leaf area correlated positively with Pn (r = 0.913, *p* < 0.01), while exhibiting a negative correlation with MDA (*p* < 0.01). A discernible negative association between MDA and assorted indicators was detected, with the most pronounced difference occurring with CAT activity (r = −0.863). There was a positive and significant interrelation between soluble protein and SOD, POD, and CAT activities along with their coding gene relative expression levels (*p* < 0.01). Leaf area and Pn were significantly associated with the activities of three enzyme three enzymes (SOD, POD, CAT) and their corresponding gene expression levels.

## 4. Discussion

The emerging field of rhizosphere growth-promoting bacteria highlights the immense potential these microorganisms hold for plant health and development, as demonstrated by numerous studies on medicinal plants. Compared to other plant growth-promoting rhizobacteria, such as phosphate-solubilizing bacteria, which break down organic phosphorus compounds [44], and potassium-solubilizing bacteria, which decompose insoluble minerals in the soil [45], nitrogen-fixing bacteria offer a distinct advantage due to their high nitrogen utilization efficiency. In this study, 15 strains of nitrogen-fixing bacteria were identified in the soil of *F. taipaiensis*. The three dominant nitrogen-fixing bacteria screened were *R. Aquatilis*, *P. chlororaphis*, and *P. stellifer*. The nitrogen-fixation efficiency of *R. aquatilis* was the highest (8.49 g/kg). *P. stellifer* was 2.57 g/kg; *P. chlororaphis* was 2.23 g/kg. Theoretically, there was a positive interaction between the nitrogen-fixation efficiency of the strain and the nitrogen-fixation capacity of the plant. For instance, a previous study has shown that inoculation of soil hosting maize with *Pseudomonas stutzeri* A1501, a diazotrophic strain capable of both biological nitrogen fixation and phytohormone production, significantly enhanced plant biomass and nitrogen content by 25.4% and 7.8%, respectively [46]. Similarly, under low-nitrogen conditions, the soil of wheat inoculated with endophytic *Paenibacillus beijingensis* BJ-18 showed an 86.1% increase in shoot dry weight, accompanied by a 1.5-fold to 91.9-fold upregulation of nitrogen uptake and metabolism-related genes, thereby improving nutrient absorption efficiency [47]. These findings collectively support the hypothesis that microbial nitrogen-fixing efficacy directly correlates with plant growth promotion and nitrogen utilization efficiency. Chao Ai et al. [48] highlighted that nitrogen-fixing bacteria not only interact with their host plants but may also establish connections with other functional bacteria, fungi, and viruses. In practical applications, nitrogen-fixing bacteria have been incorporated into microbial fertilizers to help reduce environmental pollution caused by excessive fertilizer use while simultaneously improving crop yield and quality. This dual benefit underscores the practical significance of this study.

In this study, the leaf photosynthetic characteristics of *F. taipaiensis* were analyzed. Plant growth and vitality are closely linked to nitrogen availability, as sufficient nitrogen promotes protein synthesis, which, in turn, stimulates cell division and growth [49]. Under the experimental conditions outlined in this study, growth parameters, including leaf area, leaf thickness, plant height, stem thickness, and total leaf number, showed varying degrees of improvement following inoculation with different nitrogen-fixing bacteria. The largest leaf area was observed in the N5 group, indicating that the dual-inoculation of *R. aquatilis* and *P. stellifer* significantly influenced the growth of *F. taipaiensis*. This finding is generally consistent with previously reported studies [50,51,52]. For instance, the application of microbial fertilizers has been shown to increase plant height in *F. pallidiflora* Schrenk [53], while compound microbial fertilizers containing nitrogen-fixing, phosphate-solubilizing, and potassium-solubilizing bacteria have been found to enhance the leaf area index and deepen leaf coloration [54]. In this study, combined bacterial treatments proved more effective than individual treatments, likely due to the diverse plant growth-regulating substances secreted by different bacterial strains, such as indole-3-acetic acid (IAA) and cytokinin. Additionally, plant growth is intrinsically tied to photosynthesis [55]. Chlorophyll, the primary pigment involved in photosynthesis, plays a vital role in plant development and yield [56,57]. The net photosynthetic rate (Pn) serves as a key indicator of photosynthetic activity in individual plants. The findings from this study demonstrate that inoculation with nitrogen-fixing bacteria positively influenced chlorophyll, carotenoid, Pn, Cond, Ci, and Tr in *F. taipaiensis* leaves. This effect may be attributed to sufficient nitrogen availability, which provides essential raw materials for chlorophyll and carotenoid synthesis. The resulting increase in pigment content enhances light absorption and conversion efficiency, ultimately raising Pn. These results align with the findings of Kaichao Wu et al. [58], who reported that inoculating sugarcane seedlings of different genotypes with the endogenous nitrogen-fixing bacterium Pantoea agglomerans led to increased chlorophyll a content and Pn during the elongation phase, surpassing the control group. Similarly, Shunyi Zheng et al. [59] found that inoculation with arbuscular mycorrhizal fungi improved Pn, Cond, and Tr in pepper leaves.

Nitrogen-fixing bacteria can enhance the stress resistance of *F. taipaiensis*. Under adverse conditions, malondialdehyde (MDA), a terminal product of membrane lipid peroxidation, serves as an indicator of cell membrane damage [60]. Intracellular compounds such as soluble sugars, soluble proteins, and proline, which are essential to cellular metabolism, contribute to plant resilience and survival under stress. This study demonstrated that various treatments significantly reduced MDA concentrations while increasing soluble protein and proline levels, findings that align with previous research [61]. In the dual-inoculation N5 treatment group, MDA levels exhibited a maximum reduction of 38.24% compared to the CK group, while soluble protein content increased by 113.45%. Conversely, in the tri-inoculation N7 treatment group, MDA levels, soluble sugar content, and soluble protein content remained comparable to those observed in the single-inoculation treatment group. Thus, we speculate that *R. Aquatilis*, *P. Chlororaphis*, and *P. stellifer* had a certain degree of antagonism or are related to the number of highly efficient nitrogen-fixing bacteria, which also explains why the enhancement effect on MDA, soluble protein, and proline was not as expected in the N7 group. In summary, combined bacterial inoculation demonstrates superior efficacy compared to single-inoculation treatment groups. This enhanced efficacy may arise from the synergistic effects of dual-strain co-inoculation, which likely induces systemic resistance in plants through distinct mechanisms, thereby improving their stress tolerance.

Antioxidant enzymes such as superoxide dismutase (SOD), peroxidase (POD), and catalase (CAT) play a vital role in preventing the accumulation of reactive oxygen species (O_2_·^−^ and H_2_O_2_), thereby reducing the risk of cell membrane peroxidation [62]. In this study, all treatments significantly enhanced the activities of superoxide dismutase (SOD), peroxidase (POD), and catalase (CAT), as well as the expression levels of their corresponding genes, compared to the CK group. Furthermore, plants inoculated with dual-strain treatments demonstrated superior antioxidant enzyme activity compared to those receiving single-strain treatments, indicating that combinatorial inoculation enhances physiological robustness. These findings align with previous studies reporting synergistic interactions between microbial inoculants and host antioxidant systems [63]. Notably, the dual-inoculation N5 group exhibited the highest antioxidant enzyme activities and relative gene expression levels among all treatments, while the tri-inoculation N7 group showed enzyme activities and gene expression levels comparable to those of the single-inoculation groups. This divergence may be attributed to strain-specific interactions, such as the secretion of antibiotics or competitive metabolites that interfere with the plant growth-promoting functions of co-inoculated strains, or to a threshold effect where dual-inoculation optimally stimulates antioxidant responses, whereas tri-inoculation triggers negative feedback regulation due to overstimulation. Collectively, our results demonstrate that all bacterial treatments effectively elevate SOD, POD, and CAT activities in *F. taipaiensis* leaves, underscoring the potential of nitrogen-fixing bacterial fertilizers to enhance plant antioxidant capacity and mitigate cellular damage.

The multifunctional synthetic microbial community based on biological nitrogen fixation has demonstrated significant potential for application in plant nutrition and growth promotion. However, the extent to which nitrogen-fixing bacteria can contribute to the development of a quality evaluation system for Chinese medicinal materials remains uncertain. In this study, we confirmed that different nitrogen-fixing bacteria and their combinations influenced the photosynthetic characteristics, fundamental physiological and biochemical parameters, and protective enzyme systems of *F. taipaiensis*. Future research should prioritize elucidating the assembly dynamics of rhizosphere microbiota and their co-evolutionary interplay with host plants, particularly through integrating systems-level approaches such as metagenomics, metatranscriptomics, and plant phenomics to decipher genotype × microbiome × environment interactions. By regulating the rhizosphere microbial environment, it may be possible to establish a scientific basis for the ecological cultivation of Chinese medicinal materials, ultimately ensuring the production of high-quality medicinal products.

## 5. Conclusions

The implementation of distinct nitrogen-fixing bacteria was found to have a pronounced effect on growth indices, photosynthetic parameters, photosynthetic pigment content, soluble sugar, soluble protein, proline, antioxidant enzyme activities, and coding gene relative mRNA levels in *F. taipaiensis* leaves. Furthermore, a reduction in MDA and LS content was observed. These findings underscore that the inoculation of combined strains demonstrated superior efficacy in fostering the growth of *F. taipaiensis* compared with the inoculation of single strains. Among the combinations, the dual-inoculation of *R. aquatilis* and *P. stellifer* yielded the most favorable outcomes. Therefore, in our study, the dual-inoculation of *R. aquatilis* and *P. stellifer*, which has been developed and promoted, serves as an effective technical measure. It can not only enhance the quality of *F. taipaiensis* and promote plant growth but also improve soil fertility and boost nitrogen utilization efficiency. In addition, it can also reduce fertilizer pollution, improve soil conditions, and create favorable conditions for the further cultivation of *F. taipaiensis*, forming a virtuous cycle in artificial cultivation.

## Figures and Tables

**Table 1:** Experimental design.

Treatments	Inoculated Strain	Inoculation Measurement	Pots
N1	*Rahnella aquatilis*	120 mL/strain	10
N2	*Pseudomonas chlororaphis*	120 mL/strain	10
N3	*Paenibacillus stellifer*	120 mL/strain	10
N4	*R. Aquatilis*, *P. chlororaphis*	60 mL/strain	10
N5	*R. Aquatilis*, *P. stellifer*	60 mL/strain	10
N6	*P. Chlororaphis*, *P. stellifer*	60 mL/strain	10
N7	*R. Aquatilis*, *P. chlororaphis*, *P. stellifer*	40 mL/strain	10
CK	Replace with sterile water	120 mL sterile water	10

**Table 2:** Primer information for RT-qPCR analysis.

Genes	Forward /Reverse	Sequences	Product Size (bp)	Tm/°C
*SOD*	F:	TTCAGTTTCTTAGTGACAATAGGCG	195	58.80
	R:	GGTCTTAGTCTGGATACGGCAA		60.30
*POD*	F:	TTTCCTTTCCATTCACCCG	175	58.20
	R:	AAGACCCTTCCCTTTGTTCG		58.40
*CAT*	F:	TATTCCACAACAACGAAAGCAC	183	58.10
	R:	GGACCCGAATCCGTTAGTATG		58.10
*rpl16*	F:	TTCGTGCTACATTCGTAGGGTC	190	59.60
	R:	GTTCCATTGCGGAGTTCGG		61.00

**Table 3:** Strain growth indices and comprehensive scores.

Strains Code	Nitrogen Fixation Efficiency Value (g/kg)	Increased Indole-3-Acetic Acid Content (µg/mL)	Comprehensive Score
WXY-2	0.49 ± 0.21 ^gh^	5.85 ± 0.14 ^g^	0.113
DY-5	2.57 ± 0.30 ^cd^	10.12 ± 0.18 ^c^	0.494
DY-8	1.34 ± 0.18 ^f^	8.74 ± 0.23 ^d^	0.559
LYX-4	0.78 ± 0.01 ^gh^	6.26 ± 0.18 ^f^	0.228
WX-2	2.97 ± 0.33 ^c^	3.70 ± 0.06 ^i^	0.298
DY-7	2.41 ± 0.21 ^d^	2.13 ± 0.14 ^kl^	0.101
WXY-1	0.47 ± 0.09 ^gh^	6.38 ± 0.14 ^f^	0.493
CK-3	0.83 ± 0.06 ^g^	1.27 ± 0.18 ^m^	0.026
TB *	2.23 ± 0.11 ^d^	2.29 ± 0.16 ^j^	0.470
DY-1	0.79 ± 0.07 ^gh^	20.67 ± 0.20 ^a^	0.265
LYX-2	1.81 ± 0.14 ^e^	0.69 ± 0.16 ^n^	0.059
WX-5	8.49 ± 0.70 ^a^	7.42 ± 0.21 ^e^	0.604
CK-1	3.46 ± 0.09 ^b^	1.94 ± 0.14 ^l^	0.142
DY-2	0.38 ± 0.07 ^h^	5.11 ± 0.16 ^h^	0.055
WXY-5	3.45 ± 0.12 ^b^	14.93 ± 0.08 ^b^	0.457

Note: The data for nitrogen-fixation efficiency values and increased indole-3-acetic acid content are expressed as means ± standard deviation; *n* = 3; increased indole-3-acetic acid content compared with non-inoculated medium. Different letters in the same column indicate significant differences at *p* < 0.05 levels, as detected using the Least Significant Difference (LSD) test. Letter assignments follow a descending order of significance, where ‘a’ indicates the most pronounced difference, followed sequentially by ‘b’, with subsequent letters (c, d, etc.) representing progressively smaller magnitudes of divergence. * denotes a type of marker symbol, no functional implications.

**Table 4:** Growth indexes of *Fritillaria taipaiensis* P. Y. Li under different treatments.

Treatments	Leaf Area (cm^2^)	Leaf Thickness (mm)	Stem Diameter (mm)	Plant Height (cm)
N1	1.175 ± 0.188 ^c^	0.462 ± 0.081 ^b^	1.167 ± 0.114 ^ab^	5.488 ± 0.406 ^b^
N2	1.172 ± 0.235 ^c^	0.512 ± 0.044 ^ab^	1.040 ± 0.093 ^bc^	5.432 ± 0.389 ^b^
N3	1.163 ± 0.235 ^c^	0.527 ± 0.058 ^ab^	1.077 ± 0.147 ^abc^	5.425 ± 0.698 ^b^
N4	1.576 ± 0.274 ^b^	0.510 ± 0.042 ^ab^	1.203 ± 0.131 ^ab^	6.457 ± 0.410 ^a^
N5	1.867 ± 0.252 ^a^	0.523 ± 0.048 ^ab^	1.223 ± 0.152 ^a^	7.082 ± 0.744 ^a^
N6	1.404 ± 0.198 ^bc^	0.575 ± 0.039 ^a^	1.242 ± 0.104 ^a^	6.587 ± 0.772 ^a^
N7	1.225 ± 0.196 ^c^	0.547 ± 0.052 ^a^	1.252 ± 0.145 ^a^	4.923 ± 0.403 ^b^
CK	0.726 ± 0.068 ^d^	0.293 ± 0.062 ^c^	0.967 ± 0.164 ^c^	4.083 ± 0.485 ^c^

Note: The data are expressed as means ± standard deviation; *n* = 10. Different letters in the same column indicate significant differences at *p* < 0.05 levels, as detected using the Least Significant Difference (LSD) test. Letter assignments follow a descending order of significance, where ‘a’ indicates the most pronounced difference, followed sequentially by ‘b’, with subsequent letters (c, d, etc.) representing progressively smaller magnitudes of divergence.

**Table 5:** Photosynthetic parameters of *F. taipaiensis* leaves under different treatments.

Treatments	Net Photosynthetic Rate (Pn) Mmol/(m^2^·s)	Stomatal Conductance (Cond) μmol/(m^2^·s)	Intercellular CO_2_ Concentration (Ci) μmol/(m^2^·s)	Transpiration Rate (Tr) Mmol/(m^2^·s)	Stomatal Limitation (LS) mmol/mol
N1	10.108 ± 0.069 ^g^	0.073 ± 0.003 ^e^	148.167 ± 3.710 ^e^	1.514 ± 0.008 ^f^	0.630 ± 0.009 ^b^
N2	11.211 ± 0.008 ^d^	0.069 ± 0.006 ^ef^	146.000 ± 2.098 ^e^	1.459 ± 0.006 ^g^	0.661 ± 0.005 ^a^
N3	10.647 ± 0.233 ^f^	0.079 ± 0.004 ^d^	165.333 ± 2.805 ^c^	1.608 ± 0.074 ^d^	0.587 ± 0.007 ^d^
N4	12.459 ± 0.071 ^c^	0.084 ± 0.004 ^c^	169.667 ± 3.724 ^b^	1.775 ± 0.006 ^c^	0.576 ± 0.009 ^e^
N5	12.108 ± 0.007 ^a^	0.110 ± 0.005 ^a^	199.500 ± 3.507 ^a^	1.970 ± 0.006 ^a^	0.502 ± 0.009 ^f^
N6	11.703 ± 0.007 ^b^	0.095 ± 0.002 ^b^	157.167 ± 2.639 ^d^	1.840 ± 0.004 ^b^	0.607 ± 0.006 ^c^
N7	10.915 ± 0.016 ^e^	0.079 ± 0.003 ^d^	159.667 ± 3.386 ^d^	1.561 ± 0.003 ^e^	0.601 ± 0.008 ^c^
CK	9.326 ± 0.019 ^h^	0.068 ± 0.005 ^f^	135.833 ± 2.229 ^f^	1.388 ± 0.005 ^h^	0.635 ± 0.005 ^b^

Note: The data are expressed as means ± standard deviation; *n* = 6. Different letters in the same column indicate significant differences at *p* < 0.05 levels, as detected using the Least Significant Difference (LSD) test. Letter assignments follow a descending order of significance, where ‘a’ indicates the most pronounced difference, followed sequentially by ‘b’, with subsequent letters (c, d, etc.) representing progressively smaller magnitudes of divergence.

**Table 6:** Photosynthetic pigment levels in *F. taipaiensis* leaves under different treatments.

Treatments	Chlorophyll a (mg/g)	Chlorophyll b (mg/g)	Carotenoid (mg/g)	Chlorophyll a/b	Total Chlorophyll (mg/g)
N1	0.996 ± 0.034 ^a^	0.344 ± 0.008 ^a^	0.304 ± 0.012 ^a^	2.900 ± 0.084 ^cd^	1.339 ± 0.039 ^ab^
N2	0.965 ± 0.041 ^b^	0.339 ± 0.020 ^ab^	0.278 ± 0.037 ^b^	2.847 ± 0.095 ^cd^	1.304 ± 0.059 ^b^
N3	0.931 ± 0.011 ^c^	0.305 ± 0.006 ^c^	0.262 ± 0.038 ^bc^	3.049 ± 0.052 ^bc^	1.236 ± 0.014 ^c^
N4	0.819 ± 0.017 ^e^	0.304 ± 0.016 ^c^	0.241 ± 0.013 ^cd^	2.702 ± 0.155 ^d^	1.124 ± 0.023 ^d^
N5	1.021 ± 0.006 ^a^	0.351 ± 0.004 ^a^	0.307 ± 0.004 ^a^	2.912 ± 0.031 ^cd^	1.372 ± 0.008 ^a^
N6	1.017 ± 0.032 ^a^	0.325 ± 0.021 ^b^	0.278 ± 0.006 ^d^	3.146 ± 0.279 ^b^	1.342 ± 0.032 ^ab^
N7	0.864 ± 0.005 ^d^	0.286 ± 0.007 ^d^	0.236 ± 0.003 ^b^	3.019 ± 0.063 ^bc^	1.150 ± 0.010 ^d^
CK	0.923 ± 0.031 ^c^	0.235 ± 0.019 ^e^	0.305 ± 0.004 ^a^	3.951 ± 0.386 ^a^	1.158 ± 0.030 ^d^

Note: The data are expressed as means ± standard deviation; *n* = 6. Different letters in the same column indicate significant differences at *p* < 0.05 levels, as detected using the Least Significant Difference (LSD) test. Letter assignments follow a descending order of significance, where ‘a’ indicates the most pronounced difference, followed sequentially by ‘b’, with subsequent letters (c, d, etc.) representing progressively smaller magnitudes of divergence.

**Table 7:** MDA, soluble sugar, soluble protein, and proline content of *F. taipaiensis* leaves under different treatments.

Treatments	MDA mmol/g	Soluble Sugar mmol/g	Soluble Protein mg/g	Proline mg/g
N1	0.029 ± 0.001 ^b^	0.596 ± 0.009 ^c^	14.475 ± 0.385 ^g^	0.784 ± 0.005 ^b^
N2	0.023 ± 0.001 ^de^	0.492 ± 0.004 ^g^	18.115 ± 0.458 ^d^	0.594 ± 0.005 ^f^
N3	0.022 ± 0.001 ^de^	0.689 ± 0.008 ^a^	21.263 ± 0.608 ^c^	0.767 ± 0.058 ^c^
N4	0.029 ± 0.002 ^b^	0.430 ± 0.008 ^h^	22.603 ± 0.388 ^b^	0.750 ± 0.006 ^c^
N5	0.021 ± 0.002 ^e^	0.557 ± 0.010 ^d^	28.148 ± 0.357 ^a^	0.694 ± 0.008 ^d^
N6	0.024 ± 0.001 ^d^	0.514 ± 0.008 ^e^	17.072 ± 0.330 ^e^	0.657 ± 0.004 ^e^
N7	0.027 ± 0.001 ^c^	0.654 ± 0.011 ^b^	15.003 ± 0.374 ^f^	0.944 ± 0.007 ^a^
CK	0.034 ± 0.001 ^a^	0.503 ± 0.008 ^f^	13.187 ± 0.245 ^h^	0.550 ± 0.005 ^g^

Note: The data are expressed as means ± standard deviation; *n* = 6. Different letters in the same column indicate significant differences at *p* < 0.05 levels, as detected using the Least Significant Difference (LSD) test. Letter assignments follow a descending order of significance, where ‘a’ indicates the most pronounced difference, followed sequentially by ‘b’, with subsequent letters (c, d, etc.) representing progressively smaller magnitudes of divergence.

## Data Availability

Data from this study can be obtained from the authors upon request.

## Appendix A

**Table A1:** 16S rDNA gene sequences of strains.

Strain	16S rDNA Gene Sequences
WX-2	AACKGGCGGCMGGCTTAACaCATGCgAGTCGAgCGCCCCGCAAGGGGAGYGGCMGACGGGTGAGTAACGCGTGGGAATCTACCSTKYYCTRCGGAATAACKCMKGGAAACKKGWRYTAATACCGYATRMGCCCTWCGGGGGAAAGATTTATCGGGRAAKGATGAGCCCGCGTTGGATTAGCTAGTTGGWGGGGTAAAGGCCTACCAAGGCGACGATCCATAGCTGGTCTGASAGGATGATCARCCACATTGGGACTGARACACGGSCCAAACTCCTACGGGAGGCAGCAGTGGGGAATATTGGACAATGGGCGCAAGCCTGATCCAGCCATGCCGCGTGAGTGATGAAGGCCYTAGGGTTGTAAAGCTCTTTCACCGGTGAAGATAATGACGGTAACCGGAGAAGAAGCCCCGGCTAACTTCGTGCCARCAGCCGCGGTAATACGAAGGGGGCTAGCGTTGTTCGGAATTACTGGGCGTAAAGCGCACGTAGGCGGACATTTAAGTCAGGGGTGAAATCCCGGGGCTCAACCCCGGAACTGCCTTTGATACTGGGTGTCTAGAGTATGGAAGAGGTGAGTGGAATTCCGAGTGTAGAGGTGAAATTCGTAGATATTCGGAGGAACACCAGTGGCGAAGGCGGCTCACTGGTCCATTACTGACGCTGAGGTGCGAAAGCGTGGGGAGCAAACAGGATTAGATACCCTGGTAGTCCACGCCGTAAACGATGAATGTTAGCCGTCGGGCAGTWTACTGTTCGGTGGCGCAGCTAACGCATTAAACATTCCGCCTGGGGAGTACGGTCGCAAGATTAAAACTCAAAGGAATTGACGGGGGCCCGCACAAGCGGTGGAGCATGTGGTTTAATTYGAAGCAACGCGCAGAACCTTACCAGCCCTTGACATCCCGATTKGGGcATTacGGAGACGTTTTTCCTTCAGTTTGGCTGGATCGGAgACAGGTGSTGCATGGCTGTTGTCAGCTYGTGTYGTGAGATGTTGGGTTAAGTCCCGCAACGAGCGCAACCCTCSCCCTTAGTTGCCAGCATTTAGTTGGGCMCTYTAAGGGGACTGCCGGTGATAAGCCGAGAGGAAGGTGGGGATGACGTCAAGTCYTCATGGCCCTTACGGGYTGGGCTACACACGTGCTACAATGGTGGTGACAGTGGGCAGCGAGACMGCGAKGTCGAGCTAATCTCCAAAASCCATCTCAGTTCGGATTGCACTCTGCAACTCGAGTGCATGAAGTTGGAATCGCTAGTAATCGCAGATCAGCATGYTGCGGTGAATACGTTCCCGGGCCTTGTACACACCGCCCGTCACACCATGGGAGTTGGTTHTMCCCGAAGGTAGTGCGCTAACCGCAAGGAGGCAGCTAACCACaGKAGGTMAMGGGGGG
WX-5	CGCAGTGCGGCAGCTACACATGCAGTCGAGCGGCAGCGGAAAGTAGCTTGCTACTTTGCCGGCGAGCGGCGGACGGGTGAGTAATGTCTGGGAAACTGCCTGATGGAGGGGGATAACTACTGGAAACGGTAGCTAATACCGCATGACCTCGAAAGAGCAAAGTGGGGGATCTTCGGACCTCACGCCATCGGATGTGCCCAGATGGGATTAGCTAGTAGGTGAGGTAATGGCTCACCTAGGCGACGATCCCTAGCTGGTCTGAGAGGATGACCAGCCACACTGGAACTGAGACACGGTCCAGACTCCTACGGGAGGCAGCAGTGGGGAATATTGCACAATGGGCGCAAGCCTGATGCAGCCATGCCGCGTGTGTGAAGAAGGCCTTAGGGTTGTAAAGCACTTTCAGCGAGGAGGAAGGCATCACACTTAATACGTGTGGTGATTGACGTTACTCGCAGAAGAAGCACCGGCTAACTCCGTGCCAGCAGCCGCGGTAATACGGAGGGTGCAAGCGTTAATCGGAATTACTGGGCGTAAAGCGCACGCAGGCGGTTTGTTAAGTCAGATGTGAAATCCCCGCGCTTAACGTGGGAACTGCATTTGAAACTGGCAAGCTAGAGTCTTGTAGAGGGGGGTAGAATTCCAGGTGTAGCGGTGAAATGCGTAGAGATCTGGAGGAATACCGGTGGCGAAGGCGGCCCCCTGGACAAAGACTGACGCTCAGGTGCGAAAGCGTGGGGAGCAAACAGGATTAGATACCCTGGTAGTCCACGCTGTAAACGATGTCGACTTGGAGGTTGTGCCCTTGAGGCGTGGCTTCCGGAGCTAACGCGTTAAGTCGACCGCCTGGGGAGTACGGCCGCAAGGTTAAAACTCAAATGAATTGACGGGGGCCCGCACAAGCGGTGGAGCATGTGGTTTAATTCGATGCAACGCGAAGAACCTTACCTACTCTTGACATCCACGGAATTCGCCAGAGATGGCTTAGTGCCTTCGGGAACCGTGAGACAGGTGCTGCATGGCTGTCGTCAGCTCGTGTTGTGAAATGTTGGGTTAAGTCCCGCAACGAGCGCAACCCTTATCCTTTGTTGCCAGCACGTAATGGTGGGAACTCAAAGGAGACTGCCGGTGATAAACCGGAGGAAGGTGGGGATGACGTCAAGTCATCATGGCCCTTACGAGTAGGGCTACACACGTGCTACAATGGCATATACAAAGAGAAGCGAACTCGCGAGAGCAAGCGGACCTCATAAAGTATGTCGTAGTCCGGATTGGAGTCTGCAACTCGACTCCATGAAGTCGGAATCGCTAGTAATCGTAGATCAGAATGCTACGGTGAATACGTTCCCGGGCCTTGTACACACCGCCCGTCACACCATGGGAGTGGGTTGCAAAAGAAGTAGGTAGCTTAACCTTCGGGAGGGCGCTTACCACTTGGAATCCGGGGG
DY-1	TTGCGGGCGGGCTACACATGCAGTCGAGCGGTAGCACAGAGAGCTTGCTCTCGGGTGACGAGCGGCGGACGGGTGAGTAATGTCTGGGAAACTGCCTGATGGAGGGGGATAACTACTGGAAACGGTAGCTAATACCGCATAACGTCTCAAGACCAAAGAGGGGGACCTTCGGGCCTCTTGCCATCTCATGTGCCCAGATGGGATTAGCTAGTAGGTGGGGTAATGGGTCACCTATGCGACAATCCCTATCTGGTCTGAGAGGATGACCACCCACACTGGAACTGAGACACGGTCCACACTCCTACGGGAGGCAGCAGTGGGGAATATTGCGCAATGGGCGCAAGCCTGATGCACCCATGCCGCGTGTATGAAAAAAGCCTTCTGGTTGTAAAGTACTTTCTCCGAGGAGGAAAGCATTGTGGTTAATAACCCCAGTGATTGACGTTACTCTCACAAAAAACACCGGCTAACTCCCTGCCACCAGCCGCGGTAATACAGAGGGTGCAAGCGTTAATCTGAATTACTGGGCGTAAAGCGCACGCASGCGGTCTGTCAAGTCGGATGTGAAATCCCCGGGCTCAACCTGGGAACTGCRTTCGAAACTGGCAGGCTAKAGTCTTGTAGAGGGGGGTAGAATTCCAGGTGTAGCGGTGAAATGCGTAGAGATCTGGAGGAATACCGGTGGCGAARGCGGCCCCCTGGACAAAGACTGACRCTCASGTGCGAAAGCGTGGGGAGCAAACAGGATTAGATACCCTGGTAGTCCACGCCGTAAACGATGTCKACTTGGAGGTTGTTCCCTTGAGGAGTGGCTTCCGGAGCTAACGCGTTAAGTCGACCGCCTGGGGAGTACGGCCGCAAGGTTAAAACTCAAATGAATTGACGGGGGCCCGCACAAgCGGTGGAGCATGTGGTTTAATTCGATGCaACGCGAAGAACCTtACCTACTCTTGACATCCACGGAATTTAGCAGAGATGCTTTAGTGCCTTCGGGAACCGTGAGACAGGTGCTGCATGGCTGTCGTCAGCTCGTGTTGTGAAATGTTGGGTTAAGTCCCGCAACGAGCGCAACCCTTATCCTTTGTTGCCAGCGGTTCGGCCGGGAACTCAAAGGAGACTGCCAGTGATAAACTGGAGGAAGGTGGGGATGACGTCAAGTCATCATGGCCCTTACGAGTAGGGCTACACACGTGCTACAATGGCGCATACAAAGAGAAGCGACCTCGCGAGAGCAAGCGGACCTCATAAAGTGCGTCGTAGTCCGGATTGGAGTCTGCAACTCGACTCCATGAAGTCGGAATCGCTAGTAATCGTAGATCAGAATGCTACGGTGAATACGTTCCCGGGCCTTGTACACACCGCCCGTCACACCATGGGAGTGGGTTGCAAAAGAAGTAGGTAGCTTAACCTTCGGGAGGGCGCTTACCACTTTTGATAA
DY-2	CGCAAGGGGGGCAGCTACACATGCAGTCGAGCGGCAGCGGAAAGTAGCTTGCTACTTTGCCGGCGAGCGGCGGACGGGTGAGTAATGTCTGGGAAACTGCCTGATGGAGGGGGATAACTACTGGAAACGGTAGCTAATACCGCATGACCTCGAAAGAGCAAAGTGGGGGATCTTCGGACCTCACGCCATCGGATGTGCCCAGATGGGATTAGCTAGTAGGTGAGGTAATGGCTCACCTAGGCGACGATCCCTAGCTGGTCTGAGAGGATGACCAGCCACACTGGAACTGAGACACGGTCCAGACTCCTACGGGAGGCAGCAGTGGGGAATATTGCACAATGGGCGCAAGCCTGATGCAGCCATGCCGCGTGTGTGAAGAAGGCCTTAGGGTTGTAAAGCACTTTCAGCGAGGAGGAAGGCATCATACTTAATACGTGTGGTGATTGACGTTACTCGCAGAAGAAGCACCGGCTAACTCCGTGCCAGCAGCCGCGGTAATACGGAGGGTGCAAGCGTTAATCGGAATTACTGGGCGTAAAGCGCACGCAGGCGGTTTGTTAAGTCAGATGTGAAATCCCCGCGCTTAACGTGGGAACTGCATTTGAAACTGGCAAGCTAGAGTCTTGTAGAGGGGGGTAGAATTCCAGGTGTAGCGGTGAAATGCGTAGAGATCTGGAGGAATACCGGTGGCGAAGGCGGCCCCCTGGACAAAGACTGACGCTCAGGTGCGAAAGCGTGGGGAGCAAACAGGATTAGATACCCTGGTAGTCCACGCTGTAAACGATGTCGACTTGGAGGTTGTGCCCTTGAGGCGTGGCTTCCGGAGCTAACGCGTTAAGTCGACCGCCTGGGGAGTACGGCCGCAAGGTTAAAACTCAAATGAATTGACGGGGGCCCGCACAAGCGGTGGAGCATGTGGTTTAATTCGATGCAACGCGAAGAACCTTACCTACTCTTGACATCCACGGAATTCGCCAGAGATGGCTTAGTGCCTTCGGGAACCGTGAGACAGGTGCTGCATGGCTGTCGTCAGCTCGTGTTGTGAAATGTTGGGTTAAGTCCCGCAACGAGCGCAACCCTTATCCTTTGTTGCCAGCACGTAATGGTGGGAACTCAAAGGAGACTGCCGGTGATAAACCGGAGGAAGGTGGGGATGACGTCAAGTCATCATGGCCCTTACGAGTAGGGCTACACACGTGCTACAATGGCATATACAAAGAGAAGCGAACTCGCGAGAGCAAGCGGACCTCATAAAGTATGTCGTAGTCCGGATTGGAGTCTGCAACTCGACTCCATGAAGTCGGAATCGCTAGTAATCGTAGATCAGAATGCTACGGTGAATACGTTCCCGGGCCTTGTACACACCGCCCGTCACACCATGGGAGTGGGTTGCAAAAGAAGTAGGTAGCTTAACCTTCGGGAGGGCGCTACCACTTTGAATTCCAGG
DY-5	GAACGGGGGCGGCGTGCTATACATGCAAGTCGAGCGGAGTTATGAAGGAGCTTGCTCCGGATTAACTTAGCGGCGGACGGGTGAGTAACACGTAGGCAACCTGCCCTTCAGACTGGGATAACTACCGGAAACGGTAGCTAATACCGGATAATTCCTTTCTTCTCCTGAGGAAAGGATGAAAGACGGAGCAATCTGTTACTGAGGGATGGGCCTGCGGCGCATTAGCTAGTTGGTGGGGTAACGGCTCACCAAGGCGACGATGCGTAGCCGACCTGAGAGGGTGAACGGCCACACTGGGACTGAGACACGGCCCAGACTCCTACGGGAGGCAGCAGTAGGGAATCTTCCGCAATGGGCGAAAGCCTGACGGAGCAACGCCGCGTGAGTGATGAAGGTTTTCGGATCGTAAAGCTCTGTTGCCAGGGAAGAACGTCTCTTAGAGTAACTGCTAAAAGAGTGACGGTACCTGAGAAGAAAGCCCCGGCTAACTACGTGCCAGCAGCCGCGGTAATACGTAGGGGGCAAGCGTTGTCCGGAATTATTGGGCGTAAAGCGCGCGCAGGCGGCGATTTAAGTCTGGTGTTTAAACCATGGGCTCAACCTGTGGTCGCATCGGAAACTGGATGGCTTGAGTGCAGAAGAGGAAAGTGGAATTCCACGTGTAGCGGTGAAATGCGTAGAGATGTGGAGGAACACCAGTGGCGAAGGCGACTTTCTGGGCTGTAACTGACGCTGAGGCGCGAAAGCGTGGGGAGCAAACAGGATTAGATACCCTGGTAGTCCACGCCGTAAACGATGAGTGCTAGGTGTTAGGGGTTTCGATACCCTTGGTGCCGAAGTTAACACAGTAAGCACTCCGCCTGGGGAGTACGGTCGCAAGACTGAAACTCAAAGGAATTGACGGGGACCCGCACAAGCAGTGGAGTATGTGGTTTAATTCGAAGCAACGCGAAGAACCTTACCAGGTCTTGACATCCCCCTGAATACGTTAGAGATAGCGTAGGCCTTCGGGACAGGGGAGACAGGTGGTGCATGGTTGTCGTCAGCTCGTGTCGTGAGATGTTGGGTTAAGTCCCGCAACGAGCGCAACCCTTGACTTTAGTTGCCAGCAGGTAAGGCTGGGCACTCTAGAGTGACTGCCGGTGACAAACCGGAGGAAGGTGGGGATGACGTCAAATCATCATGCCCCTTATGACCTGGGCTACACACGTACTACAATGGCCGGTACAACGGGAAGCGAAACCGCGAGGTGGAGCCAATCTTATAAAGCCGGTCTCAGTTCGGATTGCAGGCTGCAACTCGCCTGCATGAAGTCGGAATTGCTAGTAATCGCGGATCAGCATGCCGCGGTGAATACGTTCCCGGGTCTTGTACACACCGCCCGTCACACCACGAGAGTTTACAACACCCGAAGTCGGTGGGGTAACCCGTAAGGGAGCCAGCCGCCGAAGGTGGGTAAGATAAGT
DY-7	GAAACCTGGGCCGGGCAGGGCTTAACACATGCAAGTCGAACGCCCCGCAAGGGGAGTGGCAGACGGGTGAGTAACGCGTGGGAATCTACCGTGCCCTGCGGAATAGCTCCGGGAAACTGGAATTAATACCGCATACGCCCTACGGGGGAAAGATTTATCGGGGTATGATGAGCCCGCGTTGGATTAGCTAGTTGGTGGGGTAAAGGCCTACCAAGGCGACGATCCATAGCTGGTCTGAGAGGATGATCAGCCACATTGGGACTGAGACACGGCCCAAACTCCTACGGGAGGCAGCAGTGGGGAATATTGGACAATGGGCGCAAGCCTGATCCAGCCATGCCGCGTGAGTGATGAAGGCCTTAGGGTTGTAAAGCTCTTTCACCGGAGAAGATAATGACGGTATCCGGAGAAGAAGCCCCGGCTAACTTCGTGCCAGCAGCCGCGGTAATACGAAGGGGGCTAGCGTTGTTCGGAATTACTGGGCGTAAAGCGCACGTAGGCGGATATTTAAGTCAGGGGTGAAATCCCAGAGCTCAACTCTGGAACTGCCTTTGATACTGGGTATCTTGAGTATGGAAGAGGTAAGTGGAATTCCGAGTGTAGAGGTGAAATTCGTAGATATTCGGAGGAACACCAGTGGCGAAGGCGGCTTACTGGTCCATTACTGACGCTGAGGTGCGAAAGCGTGGGGAGCAAACAGGATTAGATACCCTGGTAGTCCACGCCGTAAACGATGAATGTTAGCCGTCGGGCAGTATACTGTTCGGTGGCGCAGCTAACGCATTAAACATTCCGCCTGGGGAGTACGGTCGCAAGATTAAAACTCAAAGGAATTGACGGGGGCCCGCACAAGCGGTGGAGCATGTGGTTTAATTCGAAGCAACGCGCAGAACCTTACCAGCTCTTGACATTCGGGGTTTGGGCAGTGGAGACATTGTCCTTCAGTTAGGCTGGCCCCAGAACAGGTGCTGCATGGCTGTCGTCAGCTCGTGTCGTGAGATGTTGGGTTAAGTCCCGCAACGAGCGCAACCCTCGCCCTTAGTTGCCAGCATTTAGTTGGGCACTCTAAGGGGACTGCCGGTGATAAGCCGAGAGGAAGGTGGGGATGACGTCAAGTCCTCATGGCCCTTACGGGCTGGGCTACACACGTGCTACAATGGTGGTGACAGTGGGCAGCGAGACAGCGATGTCGAGCTAATCTCCAAAAGCCATCTCAGTTCGGATTGCACTCTGCAACTCGAGTGCATGAAGTTGGAATCGCTAGTAATCGCAGATCAGCATGCTGCGGTGAATACGTTCCCGGGCCTTGTACACACCGCCCGTCACACCATGGGAGTTGGTTTTACCCGAAGGTAGTGCGCTAACCGCAAGGAGGCAGCTAACCACGTAGTCAGGCGGTGTT
DY-8	GATGCCCCTTGCCGGGGAGGCTACACATGCAAGCCGAGCGGTAGAGGTCCTTCGGGACCTTGAGAGCGGCGCACCCGGTGCGGAACACGTGTGCAACCTGCCTTTATCTGGGGGATAGCCTTTCGAAAGGAAGATTAATACCCCATAATATATTGAATGGCATCATTTGATATTGAAAACTCCGGTGGATAGAGATGGGCACGCGCAAGATTAGATAGTTGGTAGGGTAACGGCCTACCAAGTCGATGATCTTTAGGGGGCCTGAGAGGGTGATCCCCCACACTGGTACTGAGACACGGACCAGACTCCTACGGGAGGCAGCAGTGAGGAATATTGGACAATGGGTGAGAGCCTGATCCAGCCATCCCGCGTGAAGGACGACGGCCCTATGGGTTGTAAACTTCTTTTGTACAGGGATAAACCTTTCCACGTGTGGAAAGCTGAAGGTACTGTACGAATAAGCACCGGCTAACTCCGTGCCAGCAGCCGCGGTAATACGGAGGGTGCAAGCGTTATCCGGATTTATTGGGTTTAAAGGGTCCGTAGGCGGGCCTGTAAGTCAGTGGTGAAATCTCATAGCTCAACTATGAAACTGCCATTGATACTGCAGGCCTTGAGTAGATTTGAAGTGGCTGGAATAAGTAGTGTAGCGGTGAAATGCATAGATATTACTTAGAACACCAATTGCGAAGGCAGGTCACTAAGATCTAACTGACGCTGATGGACGAAAGCGTGGGGAGCGAACAGGATTAGATACCCTGGTAGTCCACGCCGTAAACGATGCTAACTCGTTTTTGGGCTTTCGGGCTCAGAGACTAAGCGAAAGTGATAAGTTAGCCACCTGGGGAGTACGTTCGCAAGAATGAAACTCAAAGGAATTGACGGGGGCCCGCACAAGCGGTGGATTATGTGGTTTAATTCGATGATACGCGAGGAACCTTACCAAGACTTAAATGGGAAATGACAGATTTAGAAATAGATCCTTCTTCGGACATTTTCCAAGGTGCTGCATGGTTGTCGTCAGCTCGTGCCGTGAGGTGTTAGGTTAAGTCCTGCAACGAGCGCAACCCCTGTCACTAGTTGCTACCATTAAGTTGAGGACTCTAGTGAGACTGCCTACGCAAGTAGAGAGGAAGGTGGGGATGACGTCAAATCATCACGGCCCTTACGTCTTGGGCCACACACGTAATACAATGGCCGGTACAGAGGGCAGCTACACAGCGATGTGATGCAAATCTCGAAAGCCGGTCTCAGTTCGGATTGGAGTCTGCAACTCGACTCTATGAAGCTGGAATCGCTAGTAATCGCGCATCAGCCATGGCGCGGTGAATACGTTCCCGGGCCTTGTACACACCGCCCGTCAAGCCATGGAAGTCTGGGGTACCTGAAGTCGGTGACCGTAACAGGAGCTGCCTAGGTTAAACCAAGGAATCGT
CK-1	CGCAGTGGGGCAGCTACACATGCAGTCGAGCGGCAGCGGAAAGTAGCTTGCTACTTTGCCGGCGAGCGGCGGACGGGTGAGTAATGTCTGGGAAACTGCCTGATGGAGGGGGATAACTACTGGAAACGGTAGCTAATACCGCATGACCTCGAAAGAGCAAAGTGGGGGATCTTCGGACCTCACGCCATCGGATGTGCCCAGATGGGATTAGCTAGTAGGTGAGGTAATGGCTCACCTAGGCGACGATCCCTAGCTGGTCTGAGAGGATGACCAGCCACACTGGAACTGAGACACGGTCCAGACTCCTACGGGAGGCAGCAGTGGGGAATATTGCACAATGGGCGCAAGCCTGATGCAGCCATGCCGCGTGTGTGAAGAAGGCCTTAGGGTTGTAAAGCACTTTCAGCGAGGAGGAAGGCATCACACTTAATACGTGTGGTGATTGACGTTACTCGCAGAAGAAGCACCGGCTAACTCCGTGCCAGCAGCCGCGGTAATACGGAGGGTGCAAGCGTTAATCGGAATTACTGGGCGTAAAGCGCACGCAGGCGGTTTGTTAAGTCAGATGTGAAATCCCCGCGCTTAACGTGGGAACTGCATTTGAAACTGGCAAGCTAGAGTCTTGTAGAGGGGGGTAGAATTCCAGGTGTAGCGGTGAAATGCGTAGAGATCTGGAGGAATACCGGTGGCGAAGGCGGCCCCCTGGACAAAGACTGACGCTCAGGTGCGAAAGCGTGGGGAGCAAACAGGATTAGATACCCTGGTAGTCCACGCTGTAAACGATGTCGACTTGGAGGTTGTGCCCTTGAGGCGTGGCTTCCGGAGCTAACGCGTTAAGTCGACCGCCTGGGGAGTACGGCCGCAAGGTTAAAACTCAAATGAATTGACGGGGGCCCGCACAAGCGGTGGAGCATGTGGTTTAATTCGATGCAACGCGAAGAACCTTACCTACTCTTGACATCCACGGAATTCGCCAGAGATGGCTTAGTGCCTTCGGGAACCGTGAGACAGGTGCTGCATGGCTGTCGTCAGCTCGTGTTGTGAAATGTTGGGTTAAGTCCCGCAACGAGCGCAACCCTTATCCTTTGTTGCCAGCACGTAATGGTGGGAACTCAAAGGAGACTGCCGGTGATAAACCGGAGGAAGGTGGGGATGACGTCAAGTCATCATGGCCCTTACGAGTAGGGCTACACACGTGCTACAATGGCATATACAAAGAGAAGCGAACTCGCGAGAGCAAGCGGACCTCATAAAGTATGTCGTAGTCCGGATTGGAGTCTGCAACTCGACTCCATGAAGTCGGAATCGCTAGTAATCGTAGATCAGAATGCTACGGTGAATACGTTCCCGGGCCTTGTACACACCGCCCGTCACACCATGGGAGTGGGTTGCAAAAGAAGTAGGTAGCTTAACCTTCGGGAGGGCGCTACCACCTTTGATTACCGG
CK-3	GTAGTTGCGGCATGCTTAACATGCAGTCGAACGGTAACGCGGGGCAACCTGGCGACGAGTGGCGAACGGGTGAGTAATATATCGGAACGTGCCCAGTTGTGGGGGATAACTGCTCGAAAGAGCAGCTAATACCGCATACGACCTGAGGGTGAAAGCGGGGGATCGCAAGACCTCGCGCAATTGGAGCGGCCGATATCAGATTAGGTAGTTGGTGGGGTAAAGGCCTACCAAGCCGACGATCTGTAGCTGGTCTGAGAGGACGACCAGCCACACTGGGACTGAGACACGGCCCAGACTCCTACGGGAGGCAGCAGTGGGGAATTTTGGACAATGGGGGCAACCCTGATCCAGCCATGCCGCGTGCGGGAAGAAGGCCTTCGGGTTGTAAACCGCTTTTGTCAGGGAAGAAAAGACTCCTACTAATACTGGGGGTTCATGACGGTACCTGAAGAATAAGCACCGGCTAACTACGTGCCAGCAGCCGCGGTAATACGTAGGGTGCAAGCGTTAATCGGAATTACTGGGCGTAAAGCGTGCGCAGGCGGTTATGCAAGACAGATGTGAAATCCCCGGGCTCAACCTGGGAACTGCATTTGTGACTGCATGGCTAGAGTACGGTAGAGGGGGATGGAATTCCGCGTGTAGCAGTGAAATGCGTAGATATGCGGAGGAACACCGATGGCGAAGGCAATCCCCTGGACCTGTACTGACGCTCATGCACGAAAGCGTGGGGAGCAAACAGGATTAGATACCCTGGTAGTCCACGCCCTAAACGATGTCAACTGGTTGTTGGGAGGGTTTCTTCTCAGTAACGTAGCTAACGCGTGAAGTTGACCGCCTGGGGAGTACGGCCGCAAGGTTGAAACTCAAAGGAATTGACGGGGACCCGCACAAGCGGTGGATGATGTGGTTTAATTCGATGCAACGCGAAAAACCTTACCTACCCTTGACATGCCAGGAATCCTGCAGAGATGTGGGAGTGCTCGAAAGAGAACCTGGACACAGGTGCTGCATGGCCGTCGTCAGCTCGTGTCGTGAGATGTTGGGTTAAGTCCCGCAACGAGCGCAACCCTTGTCATTAGTTGCTACGAAAGGGCACTCTAATGAGACTGCCGGTGACAAACCGGAGGAAGGTGGGGATGACGTCAGGTCATCATGGCCCTTATGGGTAGGGCTACACACGTCATACAATGGCCGGGACAGAGGGCTGCCAACCCGCGAGGGGGAGCTAATCCCAGAAACCCGGTCGTAGTCCGGATCGCAGTCTGCAACTCGACTGCGTGAAGTCGGAATCGCTAGTAATCGCGGATCAGCTTGCCGCGGTGAATACGTTCCCGGGTCTTGTACACACCGCCCGTCACACCATGGGAGCGGGTTCTGCCAGAAGTAGTTAGCCTAACCGCAAGGGGGGCGATACCACCGGCAGGTTCCGTTCCC
TB *	GACCTTCGGGCCTTGCGCTATCAGATGAGCCTAGGTCGGATTAGCTAATTGGTGAGGTAATGGCTCACCAAAGGAACGATCCGTAACTGGTCTGAAAGGATGATCAGTCCCTCTGGAACTGAGACACGGGCCATACTCCTACGGGAGGCCGCAGTGGGGAATATTGGACAATGGGCGAAAGCCTGATCCAGCCATGCCGCGTGTGTGAAGAAGGTCTTCGGATTGTAAAGCACTTTAAATTGGGAGGAAGGGTTGTTCATGAATACTCTGCAATTTTGACGTTACCGACTGAATAAGCTCCGAATAACTCTGTGCCAACAGCCGCGGTAATACTAAGGGTGCAAGCGTTAATCGGAATTACTGGGCGTAAAGCGCKCGTAGGTGGTTCGTTAATCTGGATGTGAAATCCCCGGGCTCAACCTGGRAACTGCATCCRAACTGGCGAGCTWKAGTATGGWACAGGGTgGTGGAATTTCCTGTGTAGCGGTGAAATGCRTAKATATAtGRAGGAACACCAGTGGCGAASGCGACCACCTGSACTGATACTGACAcTGASGTGCGAAAaGCGTGRGGAGCAAACASGATTASaTACCCTGGDVgTCCACKCCGTAWaCGATGTCAwCTAGCCKTTGGGAGCCTTGAGCTCTTAGTGKCGCAGCTAACGCATTAAGTTGACCGCCTGGGGAGTACGKCCGCAAGGWTAAAACTCAAATGAATTGACGGKGSCCCGCACAAGCGGTGGAGCWTGTGGTTtAATTMGAAGCAACgCGRAGAACCTTACCAGGCCTTCACATCCAATGAACTTTCCAGAGATGGATTGGTGCATTCGGGAACATTGAGACAGGTGCTGCATGGCTGTCGTCAGCTCGTGTCGTGAGATGTTGGGTTAAGTCCCGTAACGAGAGCACCCCTTTTCCCTAGTTACCACCACGTAATGGTGGGCACTCTAAGGAGACTGCCGGTGACAAACCGGAGGAAGGTGGGGAAGACGTCAAGTCCTCATGGCCCTTACGGCCTGGGCTACCCACGTGCTACCATGGTCGGTACAAAGGGTCACCAAGCCACGAGGTGGAGCTAATTCCATAAAACCGATCGTAGTCCGGATCGCAGTCTGCAACTCGAGTGCGTGAAGTCGGAATCGCTAGTAATCGCGAATCAGAATGTCGCGGTGAATACGTTCCCGGGCCTTGTACACACCGCCCGTCACACCATGGGAGTGGGTTGCACCAGAAGTAGTTAGTTTAACCTTAAGGAGGAACGTTTACCCCCGGTGGTTCCAGGGGGT
LYX-2	CATACTTTCCGGCGGGCCTAACACATGCAAGTCGAGCGGCAGCGGAAAGTAGCTTGCTACTTTGCCGGCGAGCGGCGGACGGGTGAGTAATGTCTGGGAAACTGCCTGATGGAGGGGGATAACTACTGGAAACGGTAGCTAATACCGCATGACCTCGAAAGAGCAAAGTGGGGGATCTTCGGACCTCACGCCATCGGATGTGCCCAGATGGGATTAGCTAGTAGGTGAGGTAATGGCTCACCTAGGCGACGATCCCTAGCTGGTCTGAGAGGATGACCAGCCACACTGGAACTGAGACACGGTCCAGACTCCTACGGGAGGCAGCAGTGGGGAATATTGCACAATGGGCGCAAGCCTGATGCAGCCATGCCGCGTGTGTGAAGAAGGCCTTAGGGTTGTAAAGCACTTTCAGCGAGGAGGAAGGCATCACACTTAATACGTGTGGTGATTGACGTTACTCGCAGAAGAAGCACCGGCTAACTCCGTGCCAGCAGCCGCGGTAATACGGAGGGTGCAAGCGTTAATCGGAATTACTGGGCGTAAAGCGCACGCAGGCGGTTTGTTAAGTCAGATGTGAAATCCCCGCGCTTAACGTGGGAACTGCATTTGAAACTGGCAAGCTAGAGTCTTGTAGAGGGGGGTAGAATTCCAGGTGTAGCGGTGAAATGCGTAGAGATCTGGAGGAATACCGGTGGCGAAGGCGGCCCCCTGGACAAAGACTGACGCTCAGGTGCGAAAGCGTGGGGAGCAAACAGGATTAGATACCCTGGTAGTCCACGCTGTAAACGATGTCGACTTGGAGGTTGTGCCCTTGAGGCGTGGCTTCCGGAGCTAACGCGTTAAGTCGACCGCCTGGGGAGTACGGCCGCAAGGTTAAAACTCAAATGAATTGACGGGGGCCCGCACAAGCGGTGGAGCATGTGGTTTAATTCGATGCAACGCGAAGAACCTTACCTACTCTTGACATCCACGGAATTCGCCAGAGATGGCTTAGTGCCTTCGGGAACCGTGAGACAGGTGCTGCATGGCTGTCGTCAGCTCGTGTTGTGAAATGTTGGGTTAAGTCCCGCAACGAGCGCAACCCTTATCCTTTGTTGCCAGCACGTAATGGTGGGAACTCAAAGGAGACTGCCGGTGATAAACCGGAGGAAGGTGGGGATGACGTCAAGTCATCATGGCCCTTACGAGTAGGGCTACACACGTGCTACAATGGCATATACAAAGAGAAGCGAACTCGCGAGAGCAAGCGGACCTCATAAAGTATGTCGTAGTCCGGATTGGAGTCTGCAACTCGACTCCATGAAGTCGGAATCGCTAGTAATCGTAGATCAGAATGCTACGGTGAATACGTTCCCGGGCCTTGTACACACCGCCCGTCACACCATGGGAGTGGGTTGCAAAAGAAGTAGGTAGCTTAACCTTCGGGAGGGCGCTTACCCACTTTGGATTCACGGGGT
LYX-4	GAATGCGGAGCTATACATGCAGTCGGACGGGATCCGTCGGAGAGCTTGCTCGAAGACGGTGAGAGTGGCGCACGGGTGCGTAACGCGTGAGCAACCTACCTCTATCAGGGGGATAGCCTCTCGAAAGAGAGATTAACACCGCATAACATATGTGACCGGCATCGGTTGGATATTAAATATTTATAGGATAGAGATGGGCTCGCGTGACATTAGCTAGTTGGTAGGGTAACGGCTTACCAAGGCGACGATGTCTAGGGGCTCTGAGAGGAGAATCCCCCACACTGGTACTGAGACACGGACCAGACTCCTACGGGAGGCAGCAGTAAGGAATATTGGTCAATGGGCGGAAGCCTGAACCAGCCATGCCGCGTGCAGGATGACTGCCCTATGGGTTGTAAACTGCTTTTGTCCAGGAATAAACCTTTCTACGTGTAGGAAGCTGAATGTACTGGAAGAATAAGGATCGGCTAACTCCGTGCCAGCAGCCGCGGTAATACGGAGGATCCGAGCGTTATCCGGATTTATTGGGTTTAAAGGGTGCGTAGGCGGCCTATTAAGTCAGGGGTGAAATACGGTGGCTCAACCATCGCAGTGCCTTTGATACTGATGGGCTTGAATCCATTTGAAGTGGGCGGAATAAGACAAGTAGCGGTGAAATGCATAGATATGTCTTAGAACTCSGATTGCGAAGSCAGYTCAYTAAGCTGGTATTGACGCTGATGCACGAAAGCGTGGGGATCGACCAGGATTAGATACCCTGGTAGTCCACGCCCTAAACGATGATAACTCGATGTTGGGGATAGACACCCAGCGTCCAAGCGAAAGCGTTAAGTTATCCACGTGGGGAGTACGCCCGCAAGGGTGAAATTAAAGGGAATTGCCGGGGCCCCGCACAAGGGGAGGACCATGGGTTTAAATTGAAGAaTCCGGGAGGACCTTTACCCGGGCTGAAAAGTTAGGAAAGGGTGCAGAGCCCCCTTGTTCCTTGGGACcAGGAAATTAGGGCTGTCATGACGTTGTtCAGTTGGTCCCGGGGGGTGTTGGGTTAAGTCCCGCAACGAGCCCACCCCTTTTGTTTATTGCCCAGCTGTTAAGGTGGGGACTCTAAACAGACTGCCTGTGCAAACAGAGAGGAAGGTGGGGACGACGTCAAGTCATCATGGCCCTTACGTCCGGGGCTACACACGTGCTACAATGGATGGTACAGCGGGCAGCTACATAGCAATATGATGCTAATCTCTAAAAGCCATTCACAGTTCGGATTGGGGTCTGCAACTCGACCCCATGAAGTTGGATTCGCTAGTAATCGCGTATCAGCAATGACGCGGTGAATACGTTCCCGGGCCTTGTACACACCGCCCGTCAAGCCATGAAAGTTGGGGGTACCTAAAGCATGTTACCGCAAGGAGCGTGTAGGTAAGCCCAG
WXY-1	GACCATTTGGCCGGGAGGGCCTTAACACATGCAAGTCGAACGCCCCGCAAGGGGAGTGGCAGACGGGTGAGTAACGCGTGGGAATCTACCGTGCCCTGCGGAATAGCTCCGGGAAACTGGAATTAATACCGCATACGCCCTACGGGGGAAAGATTTATCGGGGTATGATGAGCCCGCGTTGGATTAGCTAGTTGGTGGGGTAAAGGCCTACCAAGGCGACGATCCATAGCTGGTCTGAGAGGATGATCAGCCACATTGGGACTGAGACACGGCCCAAACTCCTACGGGAGGCAGCAGTGGGGAATATTGGACAATGGGCGCAAGCCTGATCCAGCCATGCCGCGTGAGTGATGAAGGCCTTAGGGTTGTAAAGCTCTTTCACCGGAGAAGATAATGACGGTATCCGGAGAAGAAGCCCCGGCTAACTTCGTGCCAGCAGCCGCGGTAATACGAAGGGGGCTAGCGTTGTTCGGAATTACTGGGCGTAAAGCGCACGTAGGCGGATATTTAAGTCAGGGGTGAAATCCCAGAGCTCAACTCTGGAACTGCCTTTGATACTGGGTATCTTGAGTATGGAAGAGGTAAGTGGAATTCCGAGTGTAGAGGTGAAATTCGTAGATATTCGGAGGAACACCAGTGGCGAAGGCGGCTTACTGGTCCATTACTGACGCTGAGGTGCGAAAGCGTGGGGAGCAAACAGGATTAGATACCCTGGTAGTCCACGCCGTAAACGATGAATGTTAGCCGTCGGGCAGTATACTGTTCGGTGGCGCAGCTAACGCATTAAACATTCCGCCTGGGGAGTACGGTCGCAAGATTAAAACTCAAAGGAATTGACGGGGGCCCGCACAAGCGGTGGAGCATGTGGTTTAATTCGAAGCAACGCGCAGAACCTTACCAGCTCTTGACATTCGGGGTTTGGGCAGTGGAGACATTGTCCTTCAGTTAGGCTGGCCCCAGAACAGGTGCTGCATGGCTGTCGTCAGCTCGTGTCGTGAGATGTTGGGTTAAGTCCCGCAACGAGCGCAACCCTCGCCCTTAGTTGCCAGCATTTAGTTGGGCACTCTAAGGGGACTGCCGGTGATAAGCCGAGAGGAAGGTGGGGATGACGTCAAGTCCTCATGGCCCTTACGGGCTGGGCTACACACGTGCTACAATGGTGGTGACAGTGGGCAGCGAGACAGCGATGTCGAGCTAATCTCCAAAAGCCATCTCAGTTCGGATTGCACTCTGCAACTCGAGTGCATGAAGTTGGAATCGCTAGTAATCGCAGATCAGCATGCTGCGGTGAATACGTTCCCGGGCCTTGTACACACCGCCCGTCACACCATGGGAGTTGGTTTTACCCGAAGGTAGTGCGCTAACCGCAAGGAGGCAGCTAACCACGGTAGGTACCGGGGGGT
WXY-2	AGGGACCTTGCGGGTTGCTTTAACCATGCAAGTCGAACGGTGAAGCCAAGCTTGCTTGGTGGATCAGTGCCGAACGGGTGAGTAACACGTGAGCAACCTGCCCTGGACTCTGGGATAAGCGCTGGAAACGGCGTCTAATACTGGATATGAGACGTGATCGCATGGTCGTGTTTGGAAAGATTTTTCGGTCTGGGATGGGCTCGCGGCCTATCAGCTTGTTGGTGAGGTAATGGCTCACCAAGGCGTCGACGGGTAGCCGGCCTGAGAGGGTGACCGGCCACACTGGGACTGAGACACGGCCCAGACTCCTACGGGAGGCAGCAGTGGGGAATATTGCACAATGGGCGAAAGCCTGATGCAGCAACGCCGCGTGAGGGATGACGGCCTTCGGGTTGTAAACCTCTTTTAGCATGGAAGAAGCGAAAGTGACGGTACCTGCAGAAAAAGCGCCGGCTAACTACGTGCCAGCAGCCGCGGTAATACGTAGGGSGCAAGCGTTATCSGGAATTAtTGGGCGTAAAGAGCTCGTAGGCGgTTTGTCGCGTCTGcTGTGaAATCCCGAGGCTCAACCTCGGGCCTGCAGTGGgTACKGGCAGACTAGAGTGCGGTAGGGGAGATTGGAATTCCTGGTGTAKCGGTGGAATGCGCAGATATCAGGAgGGAACACCGATGGCGAAGGCAGATCTCTGGGCCGTAACTGACGCTGAGGAGCGAAAGGGTGGGGAGCAAACAGGCTTAGATACCcTGGTAGTCCACCCCGTAAACGTTGGGGAACTAGTTGTGGGGGACCATTCCACGGTTTCCGTGACGCAGCTAACGCATTAAGTTCCCCGCCTGGGGAGTACGGCCGCAAGGCTAAAACTCAAAGGAATTGACGGGGACCCGCACAAGCGGCGGAGCATGCGGATTAATTCGATGCAACGCGAAGAACCTTACCAAGGCTTGACATATACGAGAACGGGCCAGAAATGGTCAACTCTTTGGACACTCGTAAACAGGTGGTGCATGGTTGTCGTCAGCTCGTGTCGTGAGATGTTGGGTTAAGTCCCGCAACGAGCGCAACCCTCGTTCTATGTTGCCAGCACGTAATGGTGGGAACTCATGGGATACTGCCGGGGTCAACTCGGAGGAAGGTGGGGATGACGTCAAATCATCATGCCCCTTATGTCTTGGGCTTCACGCATGCTACAATGGCCGGTACAAAGGGCTGCAATACCGTGAGGTGGAGCGAATCCCAAAAAGCCGGTCCCAGTTCGGATTGAGGTCTGCAACTCGACCTCATGAAGTCGGAGTCGCTAGTAATCGCAGATCAGCAACGCTGCGGTGAATACGTTCCCGGGTCTTGTACACACCGCCCGTCAAGTCATGAAAGTCGGTAACACCTGAAGCCGGTGGCCCAACCCTTGTGGGAAGGGAAGCCCCGTTTGCT
WXY-5	CGATACAAAGTGGTAGCGCCCTCCCGAAGGTTAAGCTACCTACTTCTTTTGCAACCCACTCCCATGGTGTGACGGGCGGTGTGTACAAGGCCCGGGAACGTATTCACCGTAGCATTCTGATCTACGATTACTAGCGATTCCGACTTCATGGAGTCGAGTTGCAGACTCCAATCCGGACTACGACGCACTTTATGAGGTCCGCTTGCTCTCGCGAGGTCGCTTCTCTTTGTATGCGCCATTGTAGCACGTGTGTAGCCCTACTCGTAAGGGCCATGATGACTTGACGTCATCCCCACCTTCCTCCAGTTTATCACTGGCAGTCTCCTTTGAGTTCCCGGCCGAACCGCTGGCAACAAAGGATAAGGGTTGCGCTCGTTGCGGGACTTAACCCAACATTTCACaACACGAGCTGACGACAGCCATGCAGCACcTGTCTCACGGTTCCCGAAGGCACTAAAGCATCTCTGCTAAATTCCGTGGATGTCAAGAGTAGGTAAGGTTCTTCGCGTTGCATCGAATTAAACCACATGCTCCACCGCTTGTGCGGGCCCCCGTCAATTCATTTGAGTTTTAACCTTGCGGCCGTACTCCCCAGGCGGTCGACTTAACGCGTTAGCTCCGGAAGCCACTCCTCAAGGGAACAACCTCCAAGTCGACATCGTTTACGGCGTGGACTACCAGGGTATCTAATCCTGTTTGCTCCCCACGCTTTCGCACCTGAGCGTCAGTCTTTGTCCAGGGGGCCGCCTTCGCCACCGGTATTCCTCCAGATCTCTACGCATTTCACCGCTACACCTGGAATTCTACCCCCCTCTACAAGACTCTAGCCTGCCAGTTTCGAATGCAGTTCCCAGGTTGAGCCCGGGGATTTCACATCCGACTTGACAGACCGCCTGCGTGCGCTTTACGCCCAGTAATTCCGATTAACGCTTGCACCCTCCGTATTACCGCGGCTGCTGGCACGGAGTTAGCCGGTGCTTTTTCTGCGAGTAACGTCAATCACTGTGGtTATTAACCACAATGCCTTCCTCCTCGCTGAAAGTACTTTACAaCCCGAAGgCcTtCtTCATACACGCGGCATGGCTGCATCAGgCTTGCGCCCATTGTGCAATATTCCCCACTGCTGCCTCCCGTAGGAGTCTGGACCGTGTCTCAGTTCCAGTGTGGCTGGTCATCCTCTCAGACCAGCTAGGGATCGTCGCCTAGGTGAGCCATTACCCCACCTACTAGCTAATCCCATCTGGGCACATCTGATGGCAAGAGGCCCGAAGGTCCCCCTCTTTGGTCTTGCGACGTTATGCGGTATTAGCTACCGTTTCCAGTAGTTATCCCCCTCCATCAGGCAGTTTCCCAGACATTACTCACCCGTCCGCCGCTCGTCACCCGAGAGCAAGCTCTCTGTGCTACCGCTCGACTGCATGGTAGTCTGCGC

* denotes a type of marker symbol, no functional implications.

**Table A2:** Nitrogen-fixation efficiency values of 15 nitrogen-fixing bacteria.

Strains Code	Nitrogen Concentration mg/mL	Glucose Concentration g/mL	Nitrogen Fixation Efficiency Value mg/g
WXY-2	0.032	0.064	0.49 ± 0.21 gh
DY-5	0.126	0.049	2.57 ± 0.30 cd
DY-8	0.088	0.065	1.34 ± 0.18 f
LYX-4	0.047	0.06	0.78 ± 0.01 gh
WX-2	0.066	0.022	2.97 ± 0.33 c
DY-7	0.129	0.054	2.41 ± 0.21 d
WXY-1	0.030	0.064	0.47 ± 0.09 gh
CK-3	0.049	0.059	0.83 ± 0.06 g
TB *	0.147	0.066	2.23 ± 0.11 d
DY-1	0.047	0.060	0.79 ± 0.07 gh
LYX-2	0.107	0.062	1.81 ± 0.14 e
WX-5	0.317	0.037	8.49 ± 0.70 a
CK-1	0.208	0.060	3.46 ± 0.09 b
DY-2	0.023	0.061	0.38 ± 0.07 h
WXY-5	0.232	0.067	3.45 ± 0.12 ^b^

Note: The data for nitrogen concentration and glucose concentration are expressed as means. The data for nitrogen-fixation efficiency values are expressed as means ± standard deviation. Different letters in the same column indicate significant differences at *p* < 0.05 levels, as detected using the LSD test. * denotes a type of marker symbol, no functional implications.

**Table A3:** UV absorption value with respect to CAT activity of *F. taipaiensis* leaves under different treatments.

Treatments	A_ck_	A_S_	t (min)	CAT Activity
N1	0.573	0.998	1	272.448
N2	0.576	1.226	0.5	268.145
N3	0.575	0.947	1	363.615
N4	0.570	0.886	1	281.073
N5	0.579	0.890	1	386.264
N6	0.570	0.967	1	295.869
N7	0.579	0.979	0.5	277.243
CK	0.576	1.221	0.5	187.301

Note: A_ck_ is the light absorption value of a blank control and A_S_ is the light absorption value of the sample. The data are expressed as means.

**Table A4:** Content of SOD, POD, and CAT activity of *F. taipaiensis* leaves under different treatments.

Treatments	SOD Activity	POD Activity	CAT Activity
N1	240.893 ± 22.626 ^c^	191.156 ± 14.512 ^d^	272.448 ± 9.830 ^d^
N2	165.450 ± 27.354 ^e^	175.136 ± 17.594 ^d^	268.145 ± 17.285 ^d^
N3	258.581 ± 20.496 ^c^	302.837 ± 26.645 ^c^	363.615 ± 10.146 ^b^
N4	213.118 ± 8.238 ^d^	366.403 ± 32.585 ^b^	281.073 ± 7.611 ^cd^
N5	376.226 ± 25.008 ^c^	429.907 ± 13.612 ^a^	386.264 ± 26.560 ^a^
N6	284.913 ± 10.039 ^b^	350.404 ± 26.323 ^b^	295.869 ± 22.913 ^c^
N7	243.979 ± 11.109 ^c^	302.666 ± 26.383 ^c^	277.243 ± 6.843 ^cd^
CK	156.072 ± 21.728 ^e^	164.975 ± 8.209 ^d^	187.301 ± 10.351 ^e^

Note: The data are expressed as means ± standard deviation; *n* = 6. Different letters in the same column indicate significant differences at *p* < 0.05 levels, as detected using the LSD test.

**Table A5:** Expression levels of *SOD*, *POD*, and *CAT* genes of *F. taipaiensis* leaves under different treatments.

Treatments	*SOD* Gene	*POD* Gene	*CAT* Gene
N1	2.911 ± 0.530 ^bcd^	3.349 ± 0.914 ^d^	4.724 ± 0.141 ^d^
N2	1.712 ± 0.277 ^de^	1.963 ± 0.092 ^e^	4.048 ± 0.122 ^d^
N3	3.668 ± 0.217 ^bc^	4.630 ± 0.065 ^cd^	6.781 ± 1.075 ^b^
N4	2.690 ± 0.044 ^cd^	7.335 ± 0.766 ^b^	4.899 ± 0.360 ^cd^
N5	5.722 ± 1.050 ^a^	9.212 ± 1.184 ^a^	8.794 ± 0.844 ^a^
N6	4.009 ± 0.589 ^b^	6.765 ± 0.635 ^b^	5.911 ± 0.637 ^bc^
N7	3.491 ± 1.076 ^bc^	5.400 ± 0.451 ^c^	4.643 ± 0.390 ^d^
CK	1.100 ± 0.088 ^e^	0.973 ± 0.156 ^e^	1.032 ± 0.030 ^e^

Note: The data are expressed as means ± standard deviation; *n* = 6. Different letters in the same column indicate significant differences at *p* < 0.05 levels, as detected using the LSD test.
